# Paralog analyses reveal gene duplication events and genes under positive selection in *Ixodes scapularis* and other ixodid ticks

**DOI:** 10.1186/s12864-015-2350-2

**Published:** 2016-03-16

**Authors:** Janice P. Van Zee, Jessica A. Schlueter, Shannon Schlueter, Philip Dixon, Carlos A. Brito Sierra, Catherine A. Hill

**Affiliations:** Department of Entomology, Purdue University, 901 W. State Street, West Lafayette, IN 47907-2089 USA; Department of Bioinformatics and Genomics, University of North Carolina Charlotte, 9201 University City Blvd, Charlotte, NC 28223 USA; Department of Statistics, Iowa State University, 2121 Snedecor Hall, Ames, IA 50011 USA

**Keywords:** Ixodidae, Paralog, Positive selection, *Ixodes scapularis*, *Rhipicephalus microplus*

## Abstract

**Background:**

Hard ticks (family Ixodidae) are obligatory hematophagous ectoparasites of worldwide medical and veterinary importance. The haploid genomes of multiple species of ixodid ticks exceed 1 Gbp, prompting questions regarding gene, segmental and whole genome duplication in this phyletic group. The availability of the genome assembly for the black legged tick, *Ixodes scapularis*, and transcriptome datasets for multiple species of ticks offers an opportunity to assess the contribution of gene duplication to the genome. Here we developed a bioinformatics pipeline to identify and analyze duplicated genes (paralogs) using gene models from the prostriate tick, *I. scapularis* IscaW1.1 annotation and expressed sequence tags (ESTs) from *I. scapularis* and the metastriate ticks, *Rhipicephalus microplus* (southern cattle tick), *R. appendiculatus* (brown ear tick) and *Amblyomma variegatum* (tropical bont tick).

**Results:**

Approximately 1-2 % of *I. scapularis* gene models and 2-14 % of ESTs from the four species represent duplicated genes. The ratio of non-synonymous to synonymous nucleotide substitution rates suggests ~ 25 % of duplicated genes are under positive selection pressure in each species. Analyses of synonymous substitution rates provide evidence for two duplication events in *I. scapularis* and *R. microplus* involving several hundred genes. Conservative molecular clock estimates based on synonymous substitution rates for species of *Anopheles* mosquitoes and the fruit fly, *Drosophila melanogaster*, suggest these events occurred within the last 50 MYA. Mapping of paralogs to the *I. scapularis* genome assembly supports tandem, or possibly segmental duplication events.

**Conclusions:**

The present study marks the first genome-level analyses of gene duplication for the Ixodidae and provides insights into mechanisms shaping genome evolution in this group. At least two duplication events involving hundreds of genes may have occurred independently in the lineages prostriata and metastriata, with tandem and segmental duplication the most likely mechanisms for paralog generation. Duplicated genes under positive selection pressure may be linked to emerging functions in the tick and represent important candidates for further study.

**Electronic supplementary material:**

The online version of this article (doi:10.1186/s12864-015-2350-2) contains supplementary material, which is available to authorized users.

## Background

Families Ixodidae (hard ticks) and Argasidae (soft ticks) have haploid genome sizes ranging from 1 to more than 7 Gbp and repetitive DNA represents a significant component of the genome of ixodid ticks [1–3]. Repetitive sequence that includes duplicated genes, segmental duplications, simple sequence repeats and transposable elements, can be a major source of biological variation and is recognized as an important driving force in eukaryotic evolution [4, 5]. Studies using reassociation kinetics and flow cytometry suggest repetitive sequences account for ~70 % of the genome of the black legged tick, *Ixodes scapularis*, with tandem repeats and transposable elements contributing to the estimated ~ 40 % highly and 30 % moderately repetitive fractions, respectively. This is supported by bioinformatic and cytogenetic analyses of the assembled genome [6, 7]. However, the relative contribution of other types of duplicated sequence generated via whole-genome, segmental or tandem gene duplication events is unresolved. Duplicated sequence may occur by unequal cross-over, retro-transposition or by lack of disjunction among daughter chromosomes after replication [8]. Gene conversion, horizontal transfer, and hybridization can also give rise to duplicated sequences [9]. Duplicated genes may confer advantages by increasing protein diversity through accumulation of non-synonymous mutations and alternative splicing or by changes in expression levels or spatio-temporal expression patterns [9, 10]. These genes may also serve as a buffer against deleterious mutations or as targets for retro-transposition. Evaluation of gene duplicates in ixodid ticks is fundamental in order to understand genome organization and evolution, and may ultimately aid the selection of genes for development of targeted pest control.

Segmental and whole genome duplication events have the potential to significantly increase genome size. Ribeiro et al. [10] identified multiple putative duplicated genes, also referred to as paralogs, in expressed sequence tags (ESTs) derived from the salivary glands of *I. scapularis* and proposed that segmental or whole genome duplication events may have played a significant role in the evolution of tick genomes. Subsequent studies of the sialome (proteins expressed in the salivary glands) [11–14] and mialome (proteins expressed in the midgut) [15] of hard and soft ticks and other blood feeding arthropods also suggest duplication events. Possible duplicates include genes encoding Kunitz-domain salivary proteins in *I. scapularis* and *I. ricinus* [16] and acetylcholinesterase in *Rhipicephalus microplus* [17]. Transcriptome data from the *I. scapularis* IscaW1 genome assembly [6], the first for a tick species, and tick EST projects provide opportunities to investigate gene duplication within the genome context for this phyletic group.

Here we report the first genome-level analyses of gene duplication in four species of ixodid ticks. Using a novel bioinformatics pipeline and the program Vmatch, we identified duplicated genes (pairs of paralogs with high sequence similarity) in the prostriate tick, *I. scapularis,* and the metastriate ticks *Rhipicephalus (Boophilus) microplus, R. appendiculatus* and *Amblyomma variegatum*. PAML analyses were used to calculate the substitution rate between paralogs, identify genes under positive and negative selection and predict the time of duplication events. Gene ontology analyses were performed to investigate the function of duplicated *I. scapularis* genes. Where possible, *I. scapularis* paralog pairs were mapped to the IscaW1 genome assembly. Results are discussed in terms of the implications for genome evolution in the Ixodidae.

## Results

### Tick gene model (GM) and tentative consensus (TC) datasets

Putative duplicated genes (pairs of paralogous sequences) were identified from *I. scapularis* GMs and from *I. scapularis, R. microplus, R. appendiculatus* and *A. variegatum* TC sequences. The GM and TC sequences employed in this study are summarized in Table 1. 24,925 GMs, from the IscaW1.1 automated annotation of the *I. scapularis* genome, were used for the identification of putative paralog sequences. In addition, 20,901 TCs generated from the alignment of 192,461 *I. scapularis* ESTs produced as part of the genome project and ESTs from the metastriate ticks *R. microplus*, *R. appendiculatus* and *A. variegatum,* were included in analyses.

**Table 1:** Datasets used for the identification of duplicate genes (paralog pairs) in four species of ixodid ticks

Species	Number of ESTs	Number of TCs^a^	Number of gene models^b^
*Ixodes scapularis*	192,461	20,901	24,925
*Rhipicephalus microplus*	42,512	9403	NA
*Rhipicephalus appendiculatus*	19,123	2767	NA
*Amblyomma variegatum*	3992	478	NA

^a^Tentative consensus (TC) sequences were produced by separate alignment of expressed sequence tags (ESTs) for each species; ^b^Gene 2 models from the IscaW1.1 annotation; NA not available

### Identification of gene duplicates in *I. scapularis, R. microplus, R. appendiculatus* and *A. variegatum*

Clusters of two or more paralogous sequences identified by the program Vmatch (http://www.vmatch.de) using different stringency parameters (high, medium and low levels of sequence similarity between paralog pairs) represent between 1.55-3.41 % of *I. scapularis* GMs and 7.54-22.90 % of TCs (Additional file 1: Table S1). Of these, 83.2-86.9 % of GMs and 76-87 % of TCs were clusters of exactly two genes (paralog pairs). Depending on stringency level, the combined Vmatch-PAML analyses identified 182–320 and 1006-2256 duplicate genes from the *I. scapularis* GMs and TCs, respectively (Table 2). For the metastriate species, clusters of two or more paralogs comprised 2.65-5.42 %, 3.0-7.88 % and 3.14-8.37 % of TC sequences from *R. microplus, R. appendiculatus* and *A. variegatum*, respectively (Additional file 1: Table S1) and between 69–92.5 % of TC sequences from these species represented clusters of exactly two genes. In total, the combined Vmatch-PAML analyses identified 208–358, 50–110, 12–18 duplicate genes from the smaller *R. microplus, R. appendiculatus* and *A. variegatum* EST datasets, respectively (Table 2). The paralog pairs identified in this study represented 1.2-2.3 % of the total *I. scapularis* GMs and approximately 5.67-13.8 % of the sampled transcriptome (20,901 TCs derived from 192,461 ESTs; Additional file 1: Table S2). Similarly, paralog pairs represented ~ 1.88-5.02 % of the sampled transcriptome of the metastriate species.

**Table 2:** Number of paralogs identified from gene models (GM) and tentative consensus (TC) sequences analyzed by PAML. Paralogs were identified by Vmatch using low, medium and high stringency parameters followed by PAML analysis. Paralog pairs are reported as either GM or TC pairs

Species	Total number of GMs/TCs analyzed	Vmatch stringency level		
		Low^a^	Medium^b^	High^c^
*Ixodes scapularis*	24,925	160 GM pairs	115 GM pairs	91 GM pairs
*Ixodes scapularis*	20,901	1128 TC pairs	774 TC pairs	503 TC pairs
*Rhipicephalus microplus*	9403	178 TC pairs	132 TC pairs	104 TC pairs
*Rhipicephalus appendiculatus*	2766	55 TC pairs	35 TC pairs	25 TC pairs
*Amblyomma variegatum*	478	9 TC pairs	8 TC pairs	6 TC pairs

^a^Low stringency: 75 % of the smaller TC must match ≥ 50 % of the larger TC of a paralog pair; ^b^medium stringency: 85 % of the smaller TC must match ≥ 70 % of 4 the larger TC of a paralog pair; ^**c**^high stringency: 95 % of the smaller TC must match ≥ 80 % of the larger TC of a paralog pair

### Calculation of gene duplication rates and identification of duplicated genes under positive and negative selection

#### Calculation of synonymous (Ks) and non-synonymous (Ka) distances

Between 83.2-86.9 % of paralog pairs (clusters = 2) identified from *I. scapularis* GMs were assigned synonymous and non-synonymous substitution rates by the program PAML (Additional file 1: Table S1). Similarly, 76-87 % of *I. scapularis* TC sequences and 69–92.5 % of TC sequences from the metastriate species were assigned values by PAML. Only those sequences with PAML values were included in subsequent statistical and molecular clock analyses.

Synonymous and non-synonymous substitution rates ranged between 0.15-0.41 and 0.05-0.06, respectively (Additional file 1: Table S2; Figure S1) for paralogs identified from the *I. scapularis* GMs, and from 0.08-1.1 and 0.35-0.6 for paralogs identified from the TC sequences, respectively. For the metastriate species, synonymous and non-synonymous substitution rates ranged from 0.08-0.71 and 0.03-0.09, respectively (Additional file 1: Figure S2, Table S2). Statistical analyses and molecular clock estimates were only performed using the larger Ka/Ks datasets derived from the *I. scapularis* and *R. microplus* TC sequences.

#### Identification of paralogs under positive and negative selection

**Fig. 1:**
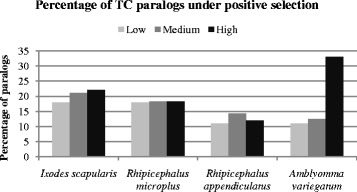
Percentage of duplicate sequences (paralog pairs) identified from the *Ixodes scapularis*, *Rhipicephalus microplus*, *R. appendiculatus* and *Amblyomma variegatum* tentative consensus (TC) sequences using Vmatch at low, medium and high stringency and under positive and negative selection based on PAML results

**Fig. 2:**
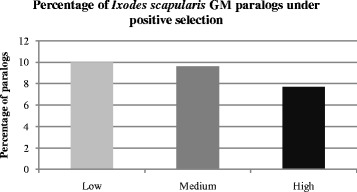
Percentage of duplicated genes (paralog pairs) in *Ixodes scapularis* identified from gene models (GMs) by VMATCH analyses at low, medium and high stringency and under positive and negative selection based on PAML analyses

Using the formula of Ka/Ks > 1, an estimated 7.8-10 % of the *I. scapularis* paralog pairs determined from GMs and 17-22 % from the TC sequences (Vmatch high, medium, low stringency parameters) may be under positive selection (Figs. 1, 2). Based on Vmatch output using high stringency parameters, 22 %, 18 %, 12 % and 33 % of the *I. scapularis*, *R. microplus*, *R. appendiculatus* and *A. variegatum* paralogs are predicted to experience positive selection (Fig. 1).

### Identification of multi-gene duplication events in *I. scapularis* and *R. microplus*

**Fig. 3:**
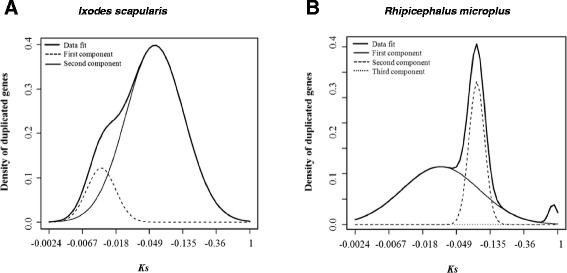
Distribution of putative duplicated sequences (paralog pairs) in (**a**) *Ixodes scapularis* and (**b**) *Rhipicephalus microplus* based on synonymous substitution rate (Ks) (Vmatch high stringency parameters) versus the density of duplicated sequences. Multi-component (first component, solid line; second component, dashed line, third component, dotted line) and best-fit (data fit, bold solid line) analyses are shown

Statistical analyses were performed on the synonymous values (Vmatch high stringency output) obtained for *I. scapularis* and *R. microplus* TC sequences to identify mixtures of normal distribution and the distribution of duplicated sequences within each mixture (Fig. 3). Equivalent analyses were not performed for *R. appendiculatus* and *A. variegatum* due to the small Ks datasets obtained for these species. Natural log (Ln) transformation of the Ks values was used to normalize standard error values. Multiple peaks indicate a mixture of normal distributions for both the *I. scapularis* and *R. microplus* paralogs. Multiple component analysis was used to determine the model which best fit the observed data. The total number of mixtures was determined based on *p* ≤ 0.05 as calculated by randomization test. Based on the probability of the entire fit to these models, the two component model was determined as the best fit for the *I. scapularis* dataset, while a three component model best fit the *R. microplus* data, where each component represents a separate duplication event involving multiple duplicate genes (i.e., sequences with similar Ks values).

### Timing of gene duplication events in *I. scapularis* and *R. microplus*

**Fig. 4:**
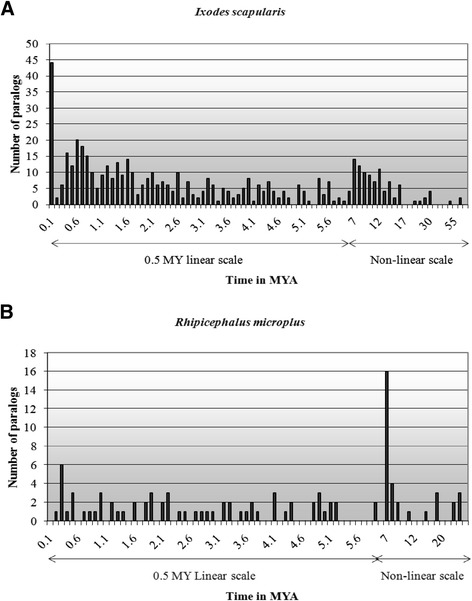
Distribution of divergence estimates for duplicated sequences from (**a**) *Ixodes scapularis* and (**b**) *Rhipicephalus microplus* based on the individual Ks rate of paralog pairs identified from TC sequences (Vmatch, high stringency) versus the number of paralogs. Data were binned in 0.1MY intervals for dates between 0–6 MY and variable (non-linear) intervals for dates > 6 MY. MYA: Million years ago

Synonymous substitutions are proposed to accumulate at a constant rate and are often used to calculate the relative age distribution of duplicated genes [18, 19]. The rates of nucleotide substitutions from several plant, animal and yeast species were used to calculate a “range” of possible dates for the duplication events in *I. scapularis* and *R. microplus* (Fig. 3, Additional file 1: Table S3). Using the *D. melanogaster* substitution rate of 16 substitutions/site/MY [20], estimates date the two *I. scapularis* gene duplication events at <1 MYA and ~5.7 MYA (Additional file 1: Table S3). The majority (>65 %) of the duplicated genes are associated with the latter event. The three putative duplication events identified for *R. microplus* date to ~ 2, 5.7 and 42.5 MYA using the *D. melanogaster* substitution rate. The majority (>85 %) of *R. microplus* paralogs are associated with the first and second events, and the remainder with a third, more ancient duplication event (Fig. 3). Substitution rates determined for a range of invertebrates, mammals, the plant *Arabidopsis thaliana* and the yeast, *Saccharomyces cerevisiae* were also used to date duplication events and reveal a wider range of estimates (Additional file 1: Table S3). To further investigate the timing and composition of duplication events, Ks rates for *I. scapularis* and *R. microplus* paralog pairs were binned into 0.1 MY intervals (for the 0–6 MYA time interval) and non-linear scale (1, 4, 5, 20 and 140 MYA intervals) for > 6MYA. The date of duplication events based on binned Ks rates for *I. scapularis* and *R. microplus* paralogs (high stringency Vmatch output) (Fig. 4) concur with dates obtained using the mean for each mixture of normal distribution.

### Functional annotation of duplicated *Ixodes scapularis* sequences

GO terms were assigned to 1276 (56 %) of the 2256 duplicated sequences identified from the *I. scapularis* TC dataset (Vmatch low stringency; Table 2) using Blast2GO [21, 22] (Additional file 1: Figures S10-S12). The analyses revealed a diverse functional annotation with 43 functional classifications identified for the “Biological Process”, 17 for the “Cellular Component” and 17 for the “Molecular Function” categories. Partitioning of data between the subsets “Positive Selection” versus “Negative Selection”, and “First Duplication Event” versus “Second Duplication Event” revealed sequences shared between subsets or assigned exclusively to one set (Additional file 1: Figures S13-S17, Tables S4, S5). Acyclic graphs and pie charts for duplicates under negative selection were supported by a greater number of GO terms than those for duplicates under positive selection (Additional file 1: Figures S13, S14, Table S4).

### Comparison of paralogs identified from *I. scapularis* GMs and TC sequences

To investigate the nature of the duplication events, reciprocal blast of the *I. scapularis* paralogs identified from GMs and TC sequences was performed to the IscaW1 supercontigs, followed by manual annotation of the corresponding loci. To compare duplicate sequences and identify those common to the GMs and TC sequences, a cross-reference file was created. Ninety five (59 %) of the 160 GMs identified as duplicates at low stringency level, matched paralogs from the TC dataset (Additional file 1: Figure S3) and five possible scenarios were determined (Additional file 1: Figure S4). For 18 cases, there was a one-to-one correlation between the number of GMs and TC sequences where two GMs mapped to separate loci in the assembly and two TC sequences mapped to these loci, indicative of tandem or segmental duplication and transcription of duplicated genes (Additional file 1: Figure S4A-C, Scenario A-C). In the majority of cases (67; 42 %) the number of TC sequences exceeded the GMs (Additional file 1: Figure S4D, Scenario D) and this may reflect the draft *I. scapularis* assembly and *ab initio* annotation. In 10 cases, the opposite was observed where the number of GMs exceeded that of the TC sequences, indicating the lack of transcripts associated with these loci and possibly reflecting either lack of transcription at the time of tissue sampling or pseudogenes. Sixty-five duplicate sequences identified from *I. scapularis* TC sequences did not match to GMs using the criteria established in this study (Additional file 1: Figure S4E, scenario E). It was not possible to confirm or infer the nature of the duplication event in the case of scenarios D and E.

## Discussion

We describe the first genome and transcriptome-level study to identify and analyze gene duplication in the prostriate tick, *I. scapularis* and complementary studies based on transcriptome sampling in a selection of metastriate species. Our analyses suggest that ~2-3 % of the ~25,000 *I. scapularis* GMs are duplicated genes, while ~7-22 % of the sampled transcriptome of the prostriate tick, *I. scapularis* and between 3-8 % of that from the metastriate ticks, *R. microplus, R. appendiculatus* and *A. variegatum* comprises transcripts from duplicated genes. The estimates derived from TC sequences are in range with reports of duplicated genes in the genomes of multiple organisms and suggest that a significant proportion of duplicated genes may be actively transcribed. Li (1997) [8] estimated that approximately 15 % of *Homo sapiens* genes are duplicates, while, Lynch and Conery [19] suggest that approximately 8 %, 10 % and 20 % of the gene complement of the fly (*D. melanogaster*), yeast (*S. cerevisiae*) and worm (*Caenorhabditis elegans*) comprises duplicated genes.

Synonymous substitutions (also known as silent substitutions) alter the nucleotide sequence of a codon without affecting the corresponding amino acid sequence, while non-synonymous substitutions confer a change in the amino acid. The selective pressure acting upon duplicated sequences can be inferred based on the ratio of non-synonymous (Ka) to synonymous (Ks) substitutions [23] where Ka/Ks ratios greater than 1 are considered indicative of positive selection. Previous studies in humans and mice suggest that a small portion (<5 %) of genes in these species are under positive selection [24]. Analyses of duplicated genes identified from the *I. scapularis* GMs suggest approximately 10 % are under positive selection, while the percentage inferred based on TC sequences for this species is slightly higher (range 18-21 %) and estimates based on transcriptome data for the metastriate ticks range from 10-33 %. Taken together, these results indicate a proportion of tick genes are under pressure to diverge, and that beneficial nucleotide substitutions may be retained to a greater extent in these tick species than in some other eukaryotes.

Multi-component curves fit to log Ks values for duplicated genes identified from the *I. scapularis* and *R. microplus* TC datasets (Fig. 3) revealed mixtures of normal distributions. Such a result is suggestive of one or more duplication events involving multiple genes and typically associated with segmental and/or genome-wide duplication events. The median value for each curve was used to estimate coalescent duplication events (i.e., events affecting multiple loci in the genome). At the stringency levels employed, we detected evidence for two major duplication events in the genome of *I. scapularis,* and possibly three major events in that of *R. microplus.* Binning analyses (Fig. 4) revealed tens of duplicate sequences associated with these events in both species, suggestive of tandem and/or segmental duplications. Given the modest numbers of genes involved, whole-genome duplication is an unlikely source of duplicates. Earlier genome-wide events can’t be ruled out but are beyond the detection limits of the present study.

Based on the assumption that there are no consequences for synonymous substitutions, researchers speculate that these mutations accumulate at a steady pace throughout evolution. Consequently, synonymous substitution rates (Ks values) are often used to formulate molecular clock estimates [25, 26]. Molecular clock estimates were developed in the present study to date the duplication events predicted for *I. scapularis* and *R. microplus*. The calibration of a molecular clock is typically achieved using the fossil record and geological data. Unfortunately, ticks are poorly represented in the fossil record, preventing the use of this approach here [27]. As an alternative, we calibrated molecular clocks for *I. scapularis* and *R. microplus* based on the average nucleotide substitution rates estimated from animals and a plant that have stronger fossil record support, as well as for species, including a yeast, that are supported by genome-wide estimates. Conservative calculations based on the *D. melanogaster* genome (estimated nucleotide substitution rate ~ 16 substitutions/site/MY) [20], suggest that the duplication events detected in the genomes of *I. scapularis* and *R. microplus* occurred in the last 43 MY (Additional file 1: Table S3). The *D. melanogaster* estimate is preferred as it is the only value derived from genome-wide analyses for an invertebrate. These calculations suggest that the two events detected for *I. scapularis* occurred less than 1 and ~ 5.7 MYA. Less conservative calculations employing substitution rates from multiple eukaryote species broaden estimates to less than 1 and ~ 37 MYA. Conservative calculations date the three events identified for *R. microplus* at ~ 2.1, 5.7 and 42.5 MYA, and from less than 1 to ~ 273 MYA when calculations include substitution rates from all species. The pro- and metastriate lineages likely diverged ~150-250 MYA during the Jurassic/Triassic Epoch (146–251 MYA) [28] and we speculate that the duplication events at ~5.7 MYA (Additional file 1: Table S3, event 2) occurred independently in the *I. scapularis* and *R. microplus* lineages. The third event in *R. microplus* dated conservatively at ~ 40 MYA may reflect a more ancient duplication event during the Eocene Epoch, coinciding with the radiation of mammals [29, 30]. The increased diversity of vertebrate hosts that occurred during this period may have had an important bearing on the evolution of tick-host interactions. Genes associated with newly derived functions may have conferred an advantage to ticks adapting to a life-cycle exploiting multiple mammalian hosts.

Blast2GO analyses revealed functional diversity of the *I. scapularis* TCs for duplicated genes under positive and negative selection, and assigned to the first and second duplication events (Additional file 1: Figures S13-S17, Tables S4, S5). Genes for the “Biological Process” pathways “cell death”, “behavior”, “sexual reproduction” and “DNA damage” and the “Molecular Function” pathways “transferase activity”, “lyase activity”, “molecular transducer activity”, “translation regulation” and “transcription regulation” were exclusive to the negative selection dataset (Additional file 1: Figures S13-S14, Table S4). While deeper sequencing is required, these duplicates may have accumulated synonymous substitutions and may reflect proteins associated with highly conserved functions. All 11 pathways identified in the “Positive Selection” subset for the “Molecular Function” category were also present in the negative selection subset. The genes assigned to these 11 groups likely encode for structural and non-structural proteins, including enzymes, receptors and proteins involved in transcriptional/translational regulation and host interactions (Additional file 1: Table S6), and may reflect the evolution of new functions in the tick.

Despite common gene ontology (GO) terms and pathways, the majority of TC sequences were assigned exclusively to either the first or second duplication event, suggesting conservation at the level of functional category only (Additional file 1: Figures S15-S17, Table S5). The “Biological Process” categories of “transport”, “response to stimulus”, “biopolymer metabolic process” and “cellular biosynthetic process” were common to the positive and negative selection datasets. The Biological Process categories “gene expression”, “multi-cellular organismal development”, “cellular protein metabolic process”, “cellular component organization and biogenesis”, “regulation of cellular process” and “nucleobase, nucleoside, nucleotide and nucleic acid metabolic process” were unique to the first event, while those of “macromolecule biosynthetic process”, “organelle organization and biogenesis”, “system development, transcription”, “cell development” and “cellular protein metabolic process” were unique to the second. The functional annotation provided a more detailed understanding of the *I. scapularis* transcriptome and a starting point for studies on genes of interest and tick genome evolution. Genome assemblies for metastriate ticks will facilitate future studies using ortholog-ortholog comparisons and enable investigations of duplications that occurred before and after the split between the pro- and metastriate lineages.

Fewer paralogs were identified from the *I. scapularis* GMs (293 clusters of size two with Vmatch and 160 paralog pairs with PAML analysis; low stringency level) than in the sampled transcriptome (TC dataset) (1443 clusters of size two with Vmatch and 1128 paralog pairs with PAML analysis; low stringency level) (Tables 2; Additional file 1: Table S1). Paralog pairs represent ~1-2 % of the gene complement, and this is lower than percentages reported for other organisms [21, 22]. Several factors may explain this discrepancy. It has been reported that assemblies based on whole-genome shotgun sequencing (the technique used to produce the *I. scapularis* assembly) may exclude recently duplicated genes because these sequences are interpreted as redundant or as alleles [20]. In addition, only those supercontigs larger than 10 Kb were used for *ab initio* prediction of GMs. Therefore, duplicated genes are likely under-represented in the annotation. As the TC sequences were generated from the alignment of ESTs obtained from multiple individual ticks, haplotypes and splice variants may also be over represented in the TC dataset, resulting in an inflation of predicted duplicates.

To support the prediction of duplicate sequences and explore duplication events within a genome context, a cross-reference file was developed comparing duplicates identified from the *I. scapularis* transcriptome (TC sequences) with those identified from the genome using GMs (Additional file 1: Figure S3). Paralogs identified from the TC dataset mapped to ninety-five of the 160 paralog pairs (60 %) identified by PAML (low stringency level; ≥75 % nucleotide identity) from GMs. From this file, scenarios A-D were identified (Additional file 1: Figure S4). In a very small number of cases (18) (Additional file 1: Figure S4A, scenarios A and B), TC sequences identified as duplicates mapped to two GMs in a one-to-one correlation and were anchored to the assembly via one or more supercontigs. This scenario enabled the unequivocal identification of gene duplicates. In a small percentage of cases (10; scenario C; Figure S4C), the number of GMs exceeded the number of TC paralog pairs mapped to genomic regions, where the prediction of a GM on the antisense strand confounded analyses. In 41 % of cases (67 paralog pairs), the TC duplicates mapped to a single GM (Additional file 1: Figure S4D, scenario D). Possible explanations include two loci condensed into a single model by annotation software or location of the second gene on a supercontig of <10 Kb that was excluded from the automated annotation. Improvements to the annotation of *I. scapularis* GMs and more extensive sampling of the *I. scapularis* transcriptome will help to resolve relationships between duplicates and facilitate a deeper exploration of the nature of duplication events. Mapping analyses highlight the limitations of duplicate studies based on transcriptome sampling alone and new short read technologies, while invaluable resources, have limitations as multiple contigs associated with a single locus or derived from members of a multi-gene family could confound duplicate identification. High quality GMs coupled with assemblies for metastriate species are required and would greatly advance studies of genome evolution across the Ixodida.

The approach employed in the present study likely identified paralogs associated with “recent” duplication events. The detection of older duplications is confounded by nucleotide diversity (sequence saturation) between paralogs. Vmatch and other analyses used to identify paralog pairs (clusters = two) that rely on sequence similarity are biased towards detection of more recently duplicated genes. Vmatch analyses also excluded gene families comprising multiple paralogs (clusters > 2), which may include the remnants of older duplication events, and these datasets may be an important source of sequences for further investigation. This phenomenon has been reported in studies of the yeast, *S. cerevisiae* and maize, *Zea mays*. Reports suggest *S. cerevisiae* experienced a whole genome duplication approximately 100 MYA, yet of the predicted duplicate genes, only 8 % can be identified as paralogs [31]. In *Z. mays*, a larger percentage (~72 %) of the paralogs retained in the genome following a duplication event that took place ~11 MYA, can still be identified by hybridization [32]. Sequence saturation has been suggested in evolutionary studies of soybean [33], humans [34], endosymbiotic bacteria [35] and *Drosophila* [36]. The situation is likely similar for pro- and metastriate ticks given the prediction that 85 % or more of duplicated genes for species in these lineages were produced during “recent” events (~6 MYA). Further confounding the detection of gene duplications is the fact that the mechanism of duplication may impact the retention of duplicates. The half-life of genes generated by small-scale duplications has been estimated at ~ 4 MY [19], while that of genes associated with whole genome duplication is ~ 33 MY [37]. These phenomena further support the theory that sequences identified in the present study are the products of small-scale gene duplication events.

Vmatch analyses were performed at a variety of stringency levels in an attempt to capture genes associated with recent and more ancient duplications. High stringency preferences the detection of recent duplications by restricting computational output to sequences that share a high level of nucleotide identity, while low stringency relaxes parameters to enable detection of more ancient duplication events (i.e., paralogs that have accumulated more nucleotide substitutions). When criteria were relaxed, the Vmatch program returned a modest increase in both the total number of clusters of paralogs and clusters = 2 (Additional file 1: Table S1). For example, at low stringency, we observed a 93 % increase in clusters = 2 for *I. scapularis* GM duplicates and 143 % increase in duplicates identified from TC sequences.

In an attempt to further investigate the nature of the duplications identified in this study, we used dot plots in conjunction with the Artemis comparison tool to compare genomic regions containing putative paralogs and explore scenarios A, B and E (Additional file 1: Figures S5, S6, S8). The visualization of gene architecture, coupled with sequence alignments of putative paralogs, enabled the differentiation of gene duplicates versus alleles and haplotypes. Nucleotide identity between duplicate genes was highest between exons (>85 % similarity) and did not typically extend into the introns and intergenic regions, suggesting duplicated genes rather than alleles or haplotypes. For reference, human sequences with >99.5 % identity were considered alleles and collapsed into a single locus [38].

Duplicate genes identified in the present study likely have little bearing on the total DNA content and size of tick genomes. Assuming a mean gene length in *I. scapularis* of 10,589 bp (See [6]) and 293–1443 paralog clusters (Vmatch low stringency GM and TC analysis, respectively), we estimate that duplicated genes and multi-gene families could account for at most ~ 0.3-1.4 % of the ~ 2.1 Gbp genome. These percentages most likely reflect tandem or possibly segmental duplication events. At the stringency levels employed, the present study revealed little evidence to support one or more genome-wide events. The far greater contribution to the haploid genome (~70 % of total DNA) of *I. scapularis* and *R. microplus* derives from tandem repeats and transposable elements [2, 6, 7].

Previous studies suggest functional redundancy in genes that originate from whole genome duplication events, while genes derived from small-scale duplication events are often under positive selection and associated with novel and essential functions [39]. Most genes produced by small-scale duplications are lost, but those that are retained have significant potential to contribute to innovation in the genome. The present study provides evidence for a significant percentage of duplicate sequences under positive selection in all four species of ticks examined. An important next step is to investigate the putative function of the gene products associated with these candidate duplicated genes.

## Conclusions

We report the first genome-scale analyses of duplicated genes in four ixodid ticks of medical and veterinary significance. Signatures for at least two duplication events, each involving hundreds of genes and likely derived by tandem or segmental duplication, are evident in the genomes of the black legged tick, *I. scapularis* and the cattle fever tick, *R. microplus*. Estimates suggest these events occurred within the last 40 MY and may coincide with the predicted radiation of ticks through Europe, the Americas and Africa. Interestingly, ~20-25 % of paralogs identified in all four tick species revealed evidence of positive selection and may be associated with the acquisition of new functions. Annotation revealed overlap at the functional level of *I. scapularis* duplicates associated with the first and second events and under negative and positive selection, providing for directed functional studies. These duplicates have high value for research to improve understanding of tick biology and as a source of novel targets for development of tick-selective control strategies.

## Methods

### Identification of duplicated genes in *I. scapularis, R. microplus, R. appendiculatus* and *A. variegatum*

Duplicated genes were identified from the *I. scapularis* IscaW1.1 GM dataset, downloaded on December 3^rd^, 2008 from VectorBase (https://www.vectorbase.org/). The dataset comprises 24,925 GMs predicted by automated and manual curation of the assembly [6]. Paralogs were also identified from TC sequences downloaded from the Dana-Farber Cancer Institute (DFCI) - The Gene Index Project (compbio.dfci.harvard.edu/tgi). TC sequences were produced by alignment of expressed sequence tags (ESTs) from the ticks, *I. scapularis*, *R. microplus*, *R. appendiculatus*, and *A. variegatum*. The *I. scapularis* TC set was downloaded on February 19^th^, 2008, and the *R. microplus*, *R. appendiculatus*, and *A. variegatum* sequences were downloaded on May 10^th^, 2007. The datasets analyzed in this study are summarized in Table 1.

Next, a bioinformatics pipeline was developed to identify candidate paralogs by linking several complementary algorithms. First, the *getorf* program [32] was used to identify all possible open reading frames (ORFs) for each GM and TC sequence. Next, the *longorf* Perl script was used to select the longest ORF for each sequence. Finally, the Vmatch program (http://www.vmatch.de) was used to separately perform an “all-against-all” comparison for the GM datasets and each of the TC datasets. Vmatch was used to translate the nucleotide sequence of each GM and TC into six ORFs and sequences were aligned using ClustalW [40]. Sequences with > 75 % similarity were considered “paralog pairs”. In order to detect paralog pairs reflecting more recent versus older duplication events, alignments were analyzed at three stringency levels: “low stringency”, where at least 75 % of the length of the smaller sequence was identical to ≥ 50 % of the longer sequence comprising a paralog pair; “medium stringency” with 85 % and 70 % match; and “high stringency” with 95 % and 80 % match, respectively (Additional file 1: Table S1). Reciprocal paralog alignment and minimum coverage was established as criteria for paralog identification. Sequences that did not meet these requirements were classified “singletons” and excluded from further analysis. Paralogs were subsequently organized into clusters based on nucleotide similarity. Only those clusters comprising two sequences (clusters of size 2) were selected for subsequent Phylogenetic Analysis by Maximum Likelihood (PAML) [30] analysis (Table 2 Additional file 1: Table S1, S2). Clusters comprising more than two sequences were considered multi-gene families and were excluded from analyses.

### Calculation of gene duplication rates and identification of duplicated genes under positive and negative selection

PAML was used to determine the synonymous (Ks) and non-synonymous (Ka) substitution rates for each paralog pair identified by Vmatch analysis. Subsequently, the ratio of Ka/Ks was used to identify genes under positive (Ka/Ks > 1) versus negative (Ka/Ks < 1) selection pressure as per [41] (Figs. 1, 2, Additional file 1: Figures S1, S2). Paralogs with identical coding sequences or lacking synonymous substitutions were excluded from further analysis.

### Identification of multi-gene duplication events in *I. scapularis* and *R. microplus*

Following the method of [33], statistical analyses were performed on the PAML output derived from the *I. scapularis* and *R. microplus* TC datasets (Vmatch high stringency) to identify mixtures of normal distributions and to assess the overall distribution of the duplicated sequences within each mixture. The median Ks value corresponding to each mixture was used to estimate the timing of duplication events. Briefly, synonymous substitutions and standard error (SE) values obtained from PAML analyses were transformed (natural log) to normalize standard deviation values. To facilitate graphical representation of the data, normal distributions were back-transformed. Mixtures of normal distributions were determined using Ks values larger than 0.05 and smaller than 1. The number of components (k) was chosen using the AIC (Akaike’s Information Criterion) statistic. Models considering 1 to 6 components were tested and the AIC statistic calculated (−2 log likelihood + 2 number of parameters) for each model, and the model with the smallest AIC value was chosen as the best fit. *P* values were computed using five degrees of freedom χ^2^ distribution. The model that best fit the observed data was selected based on statistical significance of *p* ≤ 0.05 (Fig. 3).

### Timing of gene duplication events in *I. scapularis* and *R. microplus*

The mean Ks substitution rates obtained with PAML analyses (Vmatch high stringency) were used to date gene duplication events. The formula r = K/2 T of [8] was used to calculate the timing of each event, where r is the rate of evolution, K is the number of substitutions per site and T is the time since divergence between two sequences. Nucleotide substitution rates from mammals (*H. sapiens* and *Mus musculus*), plants (*A. thaliana*), arthropods (*D. melanogaster, Anopheles gambiae, A. funestus*, *Ornithodoros savignyi*) and yeast (*S. cerevisiae*) were considered in order to calibrate the molecular clock for ticks and produce a “range” of dates for each event (Additional file 1: Table S3). The nucleotide substitution rates from *H. sapiens, A. thaliana, D. melanogaster* and *S. cerevisiae* were based on duplicated genes identified from genome assemblies [20]. The rate for *O. savignyi* was based on analyses of platelet aggregation inhibitor genes [42], for human and mouse on 47 protein-coding sequences [8] and for *Anopheles spp.,* on 157 cDNA and 40 ESTs sequences [43] (Additional file 1: Table S3).

### Comparison of paralogs identified from *I. scapularis* GMs and TC sequences

The *I. scapularis* genome assembly IscaW1 [6] was used to investigate gene duplication events. First, a cross-reference file was created to match duplicates identified from the GMs and TC sequences. Putative duplicated TC sequences were compared to each of the putative duplicated GMs in an “all-against-all” nucleotide similarity search. For hits with more than 75 % nucleotide identity and sequence coverage based on BLAST searches, putative function was assigned using BLAST results. Putative duplicates identified from GMs and TC sequences were aligned using ClustalW and MultAlin, and the percent nucleotide similarity was analyzed. To visualize duplications on the supercontigs of the *I. scapularis* IscaW1 assembly, dot plots (http://athena.bioc.uvic.ca/tools/JDotter) were constructed with the complete or partial sequence of the supercontigs containing putative duplicated sequences. The Artemis Comparison Tool (ACT) [44] was used to visualize the duplicated sequences within the supercontigs of interest (Additional file 1: Figures S4-S9).

### Functional annotation of duplicated *Ixodes scapularis* sequences

Functional annotations were assigned to the putatively duplicated *I. scapularis* TC nucleotide sequences identified by Vmatch-PAML analyses (low stringency parameters) using Blast2GO and default parameters (Conesa et al. 2005 [21]; https://www.blast2go.com) and classified by the GO categories biological processes, cellular component, and molecular function. Sequences identified as experiencing negative and positive selection were separately analyzed as above (Additional file 1: Figures S13, S14, Table S4) while those with greater than 75 % membership probability for the first and second duplication event were separately examined. Pie charts were generated using annotations associated with pathway termini supported by 25 or more GO terms (Additional file 1: Figures S15-S17, Table S5).

### Availability of data and materials

The datasets supporting the conclusions of this article are included within the article and its additional file.

## Additional file

Additional file 1:
**Title of data: Additional Notes, Figures and Tables.** Description of data: Additional Notes, Figures and Tables. (DOCX 2395 kb)

## Abbreviations

BLAST: basic local alignment search tool
DFCI: Dana farber cancer institute
EST: expressed sequence tag
Ka: non-synonymous substitutions
Ks: synonymous substitutions
GM: gene model
GO: gene ontology
MYA: million years ago
NCBI: National Center for Biotechnology Information
ORF: open reading frame
PAML: phylogenetic analysis by maximum likelihood
SE: standard error
TC: tentative consensus
