# Supplementary material for: Paralog analyses reveal gene duplication events and genes under positive selection in Ixodes scapularis and other ixodid ticks
Source: BMC Genomics. 2016 Mar 16;17:241. doi: 10.1186/s12864-015-2350-2 (PMC4793754; doi:10.1186/s12864-015-2350-2)
Supplement: Additional file 1: — Title of data: Additional Notes, Figures and Tables. Description of data: Additional Notes, Figures and Tables. (DOCX 2395 kb) [file 12864_2015_2350_MOESM1_ESM.docx]

**Additional File 1**

**Localization, confirmation and visualization of paralogs in *I. scapularis***

In order to illustrate the correlation observed between the *I. scapularis* TC and GM datasets, examples of different duplication scenarios are described below and their graphic representation displayed in additional file 4.

There were three classes of scenarios observed in this cross-reference file:

- 1. Scenarios identified in intersection between GM and TC datasets – Scenarios A, B, C and D.
  2. Scenario identified in the TC data set only – scenario E.
  3. Scenario identified in the GM dataset only – Scenario F.

The cross-reference file comparing the duplications in the TC and GM datasets and represented in the Venn diagram (Additional file 3) includes 18 cases of duplications that fit the scenarios described in A and B. These cases are examples of duplications identified in both TC and GM datasets that are likely the result of duplication events.

Scenario A. Paralogs common to the TC and GM datasets: Example of a tandem duplication is observed with the GMs ISCW002195, ISCW002196 and TCs TC18331 and TC23505, which have strong similarity to two distinct genomic areas of the supercontig DS652022. Manual annotation indicates that both genes are found in the reverse orientation. The area between nucleotides 13,162 and 9,043 accounts for the GM ISCW002195 and TC18331, while the second locus located between the nucleotides 42,524 and 25,079, contains the paralog sequence belonging to GM ISCW002196 and TC23505 (Additional file 4) A dot plot showing the position of the duplicates and the region of similarity within the supercontig is shown in additional file 5A. The nucleotide similarity between the TCs and GMs is shown by MultiAlign at the nucleotide level can be observed in additional file 5B.

Scenario B. A variation of scenario A in which there is a correlation between TC and GM paralog pairs but the paralogs are located on different supercontigs. An example of this was observed with GMs ISCW003519 and ISCW021427. These GMs and their corresponding TC paralogs (TC18777 and TC28543), are located on two different supercontigs (DS724436 and DS852472, respectively) (Additional files 4B, 6 and 7).

Scenario C. Multiple GMs to fewer TCs (10 cases): This scenario was often associated with very short gene model predictions (i.e., <100bp). In this specific example, the GM paralogs ISCW022649 and ISCW022652 correlated to the TC paralog pair TC18837 and TC23266. In addition a third short GM (ISCW022650) was identified within the genomic region as ISCW022649, but on the opposite DNA strand. The GM and TC paralog pairs identified in this example were found in tandem duplications within the same supercontig (Additional file 4C).

Scenario D. Multiple TCs to fewer GMs: In this scenario, a TC paralog pair was mapped to a genomic region containing only one GM. In each of these cases, the GM spanned a genomic region that encompassed both TC paralogs. 67 cases satisfied this scenario, represented by TC29499, TC23068 and ISCW007552 (Additional file 4D).

Scenario E. Duplicated TCs lacking GM paralogs (1,033 cases): In this scenario, paralog TCs were identified in a region of the genome that lacked GM predictions. For example, the TC paralogs TC17212 and TC33146 mapped to the supercontigs DS961942 and DS808492, respectively. However, no GMs were predicted in the regions of these supercontigs that exhibited similarity to the TCs (Additional files 4E, 8 and 9).

Scenario F. Duplicated GMs lacking TC paralogs (65 cases). In this case, paralog GMs were identified in a region of the genome that was not associated with duplicated TCs.

Visualization of putative duplicate sequences

In order to further investigate the duplication scenarios mentioned above, at least 50 additional paralog TC pairs were selected at random and subjected to manual annotation and visualized using the Artemis Comparison Tool (Artemis_v8.jar) and Dot plot (http://athena.bioc.uvic.ca/tools/JDotter) to investigate the level of nucleotide similarity between the coding and non-coding regions of TC and GM paralog pairs and surrounding upstream and downstream intergenic regions. Three examples of particular relevance to this study are described below:

Example 1: Tandem duplication (TC34473/TC21330) with corresponding gene models (ISCW011010/ISCW011011) on same supercontig (DS867079) – Scenario A.

The GM ISCW011010 is located between nucleotides 11302-13286 while ISCW011011 is located between nucleotides 21544 and 23937. The two TCs had high nucleotide similarity to both genomic regions. Of the 420 nucleotides shared among the TC and GM sequences, 85.02% of them were identical (Additional file 5).

Examples two and three use different TCs than those shown on additional files 4 but represent duplications that fit the description of scenarios B and E respectively. These examples are further described to illustrate the most common observed scenarios observed in our duplication analysis.

Example 2: TC duplicates (TC15123/TC15072) with corresponding gene models (ISCW014005/ISCW015172) on two supercontigs (DS980060/DS945359) – Scenario B, (Additional files 6 and 7).

The paralogs TC15123 and TC15072 showed strong nucleotide similarity to two different supercontigs, DS980060 and DS945359. The genomic regions corresponding to the TCs are associated with two GMs (ISCW015172 and ISCW014005). Additional file 6 shows a dot plot alignment between the two supercontigs. Diagonal lines in the dot plot correspond to areas where nucleotide similarity between the two supercontigs is observed, whereas blank areas correspond to gaps within the supercontigs. Additional file 6B is a comparison of the same supercontigs using the Artemis comparison tool. More detail of the alignment of the nucleotide sequence of TC15123, TC15072, ISCW014005 and ISCW015172 is shown in additional file 7.

This is an example of two regions of the genome that have a significant level of nucleotide similarity within the coding regions. This suggests that the genes associated with the TCs/GMs are not alleles or haplotypes. 741 of the 864 bases common to the coding sequences of the TCs and GMs (85.75%) were identical. The nucleotide similarity was not observed beyond the coding regions and dropped significantly in the intronic and intergenic areas. Intron sizes also differed between the two genes (Additional files 6 and 7).

Example 3: Two duplicates (TC24373/TC35396), located on separate supercontig (DS786998/DS775581) without corresponding gene models – Scenario E, Additional files 8 and 9. BLAST searches using the TC paralogs TC24373 and TC35396 showed that these sequences have high nucleotide similarity with supercontigs DS786998 and DS775581. These supercontigs are short (1198 and 1370 base pairs, respectively) and there are no associated GMs because only supercontigs larger than 10Kbp bases were used for automated GM prediction. The TC paralog sequence covered more than 90% of the supercontigs. Additional file 8 shows a comparisons between the two supercontigs based on a dot plot (A) and the Artemis comparison tool (B). High nucleotide similarity between duplicated sequences (TCs) was identified in the coding regions, but was not maintained in intronic regions. This is an example of a duplication showing an inverted repeated area between the coding sequences. The difference in intron sizes are observed in the displacement of the nucleotide sequence alignment between the two paralog TCs and the two supercontigs (Additional file 9).

**Supplemental Figure Legends**

**Figure S1. Average synonymous (Ks) and non-synonymous (Ka) substitution rates of duplicated genes (paralog pairs) identified from *Ixodes scapularis* gene models (GMs) by Vmatch (low, medium and high stringency) and PAML.**

**Figure S2. Average synonymous (Ks) and non-synonymous (Ka) substitution rates of duplicated sequences (paralog pairs) identified from *Ixodes scapularis, Rhipicephalus microplus*, *R. appendiculatus* and *Amblyomma variegatum* tentative consensus (TC) sequences by Vmatch at (A) high, (B) medium and (C) low stringency parameters) and PAML.**

**Figure S3. Venn diagram showing a comparative analysis of duplicate sequences (paralog pairs) identified from *I. scapularis* gene models (GMs) and tentative consensus (TC) sequences using Vmatch (low stringency parameters) and PAML and used to generate the cross reference file to map paralogs to the IscaW1 assenbly.** The number of paralog pairs assigned to duplication scenarios A-F are shown.

**Figure S4. Graphical representation showing duplication scenarios observed with duplicated genes (paralog pairs) identified from *Ixodes scapularis* gene models (GMs) and tentative consensus (TC) sequences. (A)** Scenario A, paralogs identified from TC sequences mapped to tandemly duplicated loci (two GMs) and anchored to a supercontig; **(B)** Scenario B, duplication showing paralogs identified from TC sequences mapped to two loci (two GMs) and anchored to supercontigs; **(C)** Scenario C, the same scenario as for B above but the identification of duplicate genes is confounded by a GM on the antisense strand; **(D)** Scenario D, paralogs identified from TC sequences mapped to a single locus (one GM); **(E)** Scenario E, paralogs identified from TC sequences anchored to separate supercontigs in regions that lack corresponding GMs. TC sequences, supercontigs and gene models are indicated using the TC, DS and ISCW prefix, respectively; NNN, gap of known size within a supercontig; Numbers below supercontigs correspond to the coordinates for mapped GMs and TC sequences.

**Figure S5.** **Visualization of a tandem duplication, Scenario A**. **(A)** The dot plot shows a tandem gene duplication in supercontig DS652022 and the diagonal indicates 100% nucleotide identity of the supercontig aligned to itself. Shorter parallel diagonal lines represent duplicated regions and similarity between the duplicated genes (gene models ISCW002195 and ISCW002196) and corresponding TC sequences (TC23505 and TC18331). Solid gray bars represent the supercontig sequence and smaller yellow boxes represent the duplicated genes. The central crosshairs show the X and Y coordinates**. (B)** Alignment of the nucleotide sequences of the duplicated genes (gene models ISCW002195 and ISCW002196) and corresponding TC18331 and TC23505 sequences. Red = identical residue; Blue = similar residue; Black = mismatched residue.

**Figure S6. Visualization of duplicated genes on separate supercontigs, Scenario B.** **(A)** The dot plot shows regions of nucleotide identify between the supercontigs DS945359 (horizontal axis) and DS980060 (vertical axis). Diagonal lines correspond to areas of high nucleotide identity between the duplicated genes (two gene models ISCW014005 and ISCW015172) and corresponding TC15123 and TC15072 sequences. Gray bars represent the supercontig sequence and colored boxes represent the position and exons of the duplicated genes. **(B)** Visualization using the Artemis comparison tool (Cutoff: 270) of the six possible open reading frames for supercontigs DS945359 (top) and DS980060 (bottom). Colored boxes within open reading frames represent the position of duplicated sequences and exons. Red bars connecting the supercontigs represent areas of similarity between predicted coding and non-coding regions as identified by manual annotation of the gene models.

**Figure S7.** **Alignment of gene models ISCW014005 and ISCW015172 associated with Scenario B where duplicated genes are located on separate supercontigs and supported by duplicate sequences TC151235 and TC15072.** Red = identical residue; Blue = similar residue; Black = mismatched residue.

**Figure S8.** **Visualization of scenario E with identification of duplicated sequences on separate supercontigs and lacking gene model support.** **(A)** The dot plot shows regions of nucleotide identity between supercontig DS786998 (horizontal axis) and DS775581 (vertical axis) and shorter diagonal lines correspond to regions of nucleotide identity between putative duplicates, TC24373 and TC35396. The dot plot reveals a large inversion as indicated by the inverted diagonal. Gray bars represent supercontigs and colored boxes represent the position of duplicated sequences. **(B)** Visualization using the Artemis comparison tool (Cutoff =1) between the duplicated regions identified in the two supercontigs. The supercontigs DS775581 (top) and DS786998 (bottom) and the six possible open reading frames are shown. The areas of similarity are represented by blue and red bars connecting the supercontigs. The inversion is indicated by red bars.

**Figure S9. Alignment of duplicated regions of the supercontigs DS786998 and DS775581 and corresponding duplicates, TC35396 and TC24373.** Red = identical residue; Blue = similar residue; Black = mismatched residue.

**Figure S10. Blast2GO functional analysis of *Ixodes scapularis* putative duplicated TC sequences for the biological process category.** Numbers in parenthesis indicate the total number of TC sequences assigned a functional annotation (minimum of five TC sequences for each GO annotation).

**Figure S11. Blast2GO functional analysis of *Ixodes scapularis* putative duplicated TC sequences for the cellular component category.** Numbers in parenthesis indicate the total number of TC sequences assigned a functional annotation (minimum of five TC sequences for each GO annotation).

**Figure S12. Blast2GO functional analysis of *Ixodes scapularis* putative duplicated TC sequences for the molecular function category.** Numbers in parenthesis indicate the total number of TC sequences assigned a functional annotation (minimum of five TC sequences for each GO annotation).

**Figure S13.** **Blast2GO functional analysis of *Ixodes scapularis* putative duplicated TC sequences under negative selection** **showing results for the molecular function category.** Numbers in parenthesis indicate the total number of TC sequences assigned a functional annotation (minimum of five TC sequences for each GO annotation).

**Figure S14. Blast2GO functional analysis of *Ixodes scapularis* putative duplicated TC sequences under positive selection showing results for the molecular function category.** Numbers in parenthesis indicate the total number of TC sequences assigned a functional annotation (minimum of five TC sequences for each GO annotation).

**Figure S15. Blast2GO functional analysis of *Ixodes scapularis* TC sequences assigned to the (A) first and (B) second duplication events and** **showing results for the biological process category.** Numbers in parenthesis indicate the total number of TC sequences assigned a functional annotation (minimum of 25 TC sequences for each GO annotation).

**Figure S16.** **Blast2GO functional analysis of *Ixodes scapularis* TC sequences assigned to the (A) first and (B) second duplication events and** **showing results for the cellular component category.** Numbers in parenthesis indicate the total number of TC sequences assigned a functional annotation (minimum of 25 TC sequences for each GO annotation).

**Figure S17. Blast2GO functional analysis of *Ixodes scapularis* TC sequences assigned to the (A) first and (B) second duplication events and** **showing results for the molecular function category.** Numbers in parenthesis indicate the total number of TC sequences assigned a functional annotation (minimum of 25 TC sequences for each GO annotation).


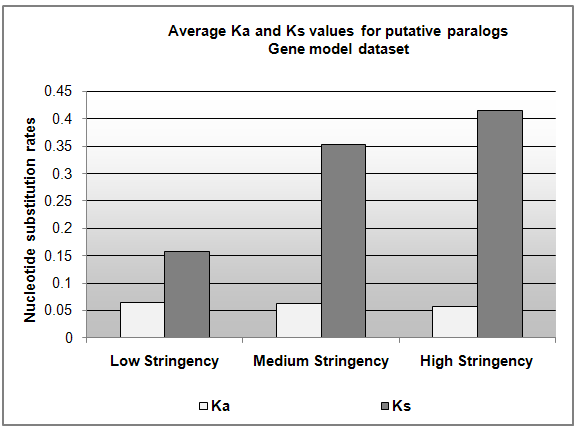


**Figure S1.**

50


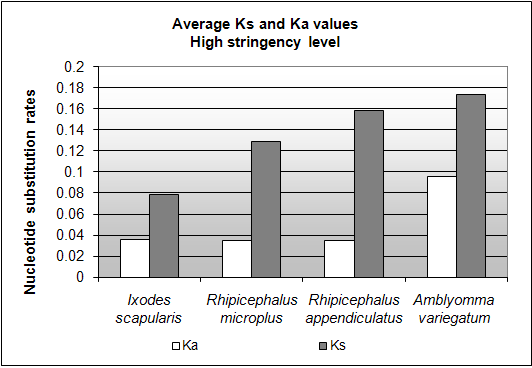


A


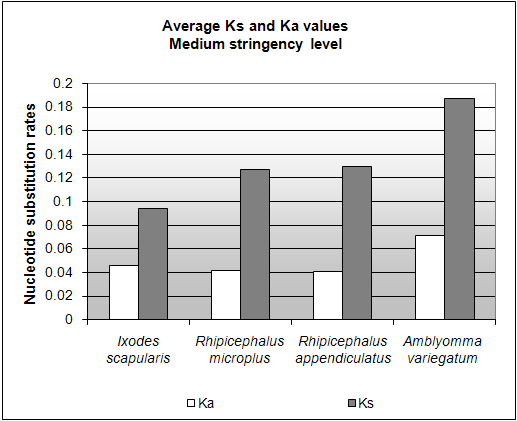

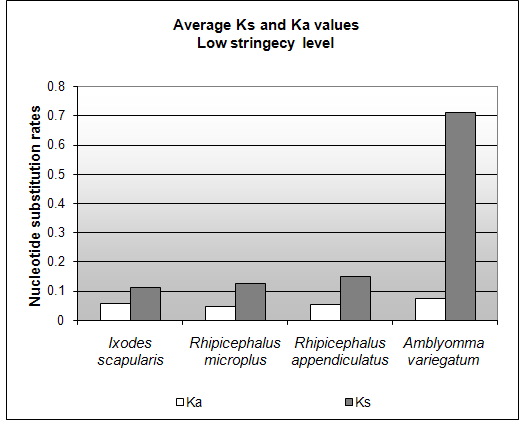


B

C

**Figure S2.**


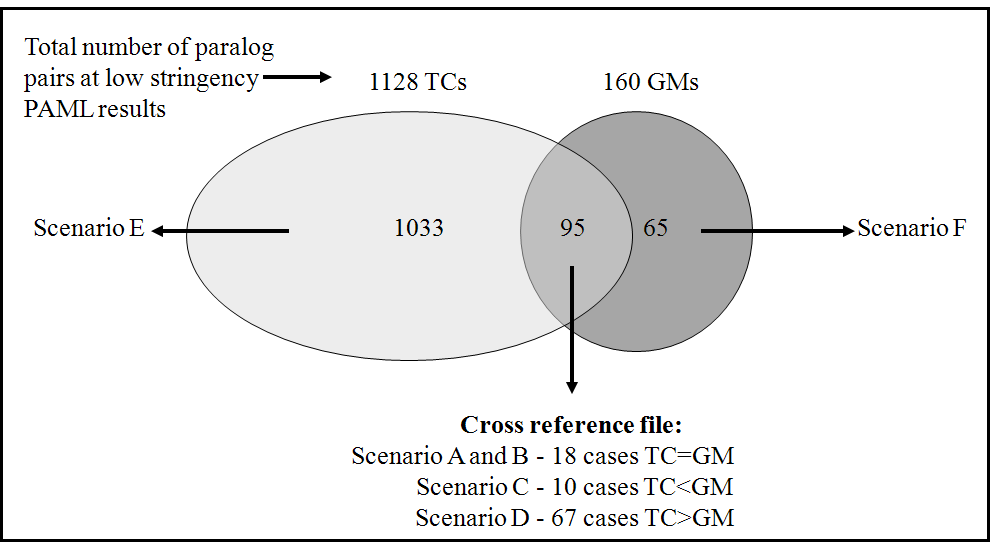


**Figure S3.**


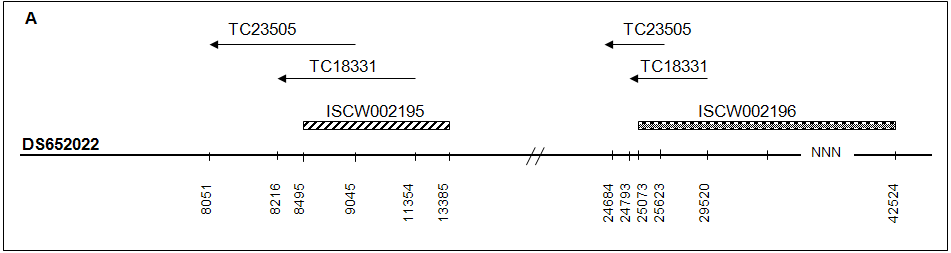

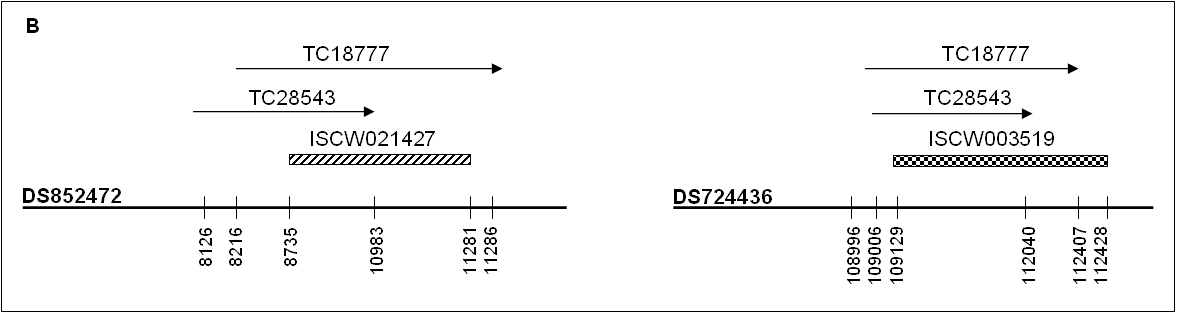


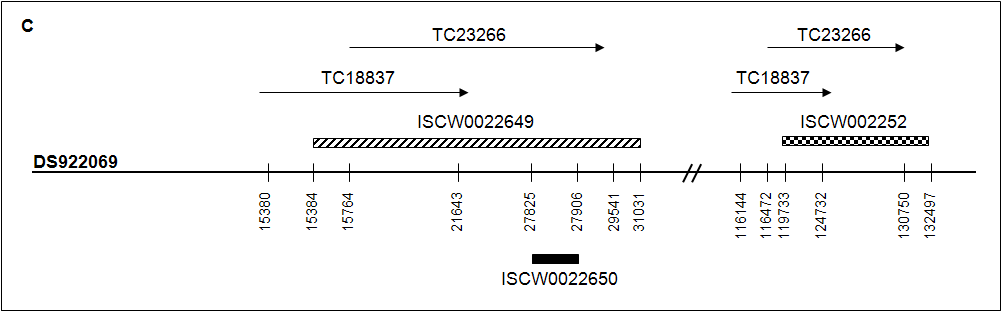

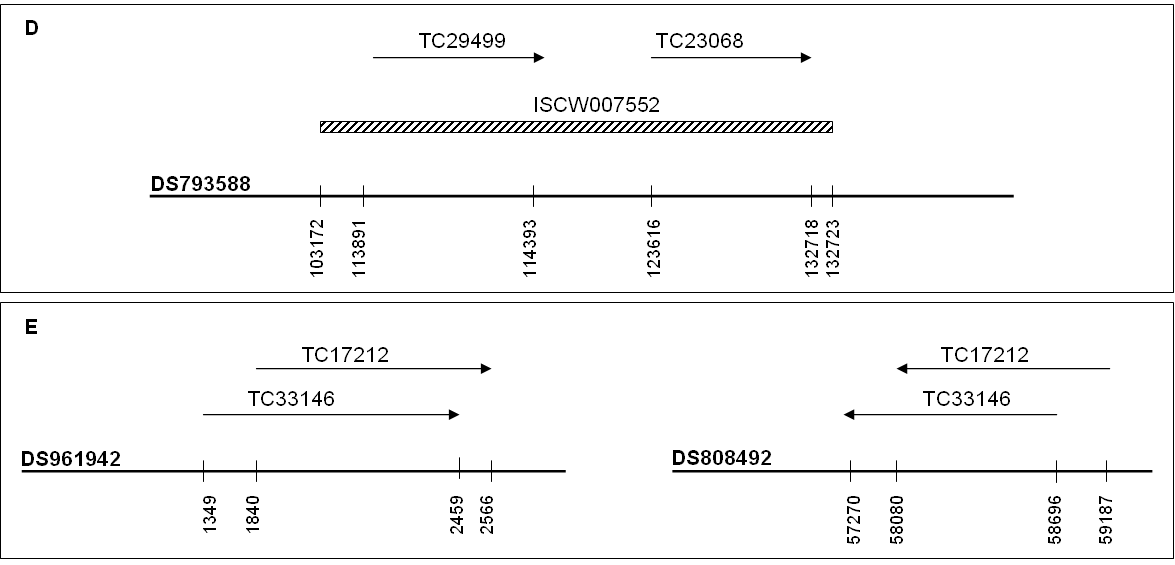


**Figure S4.**

**A. B.**


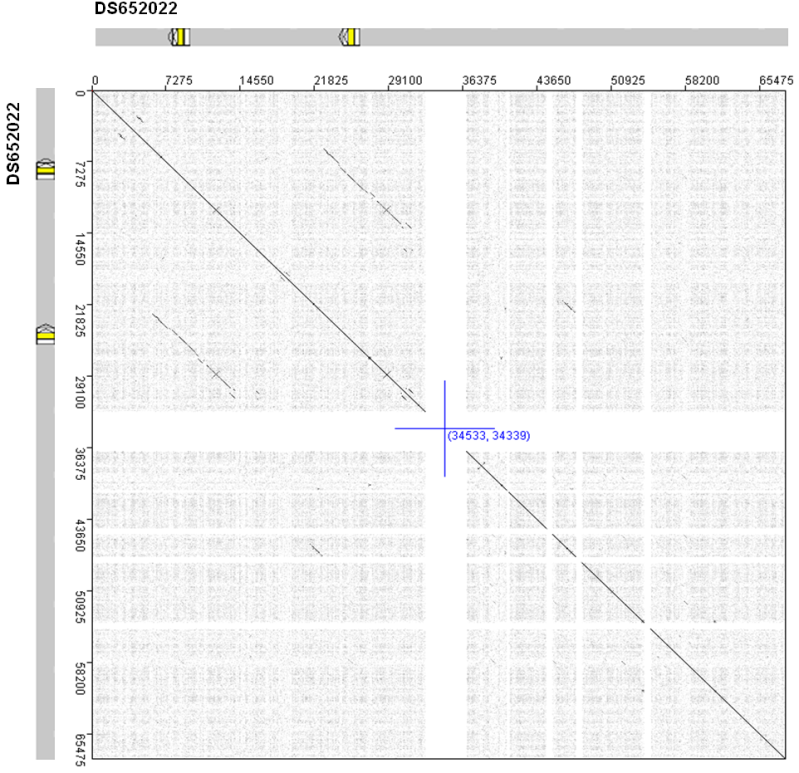

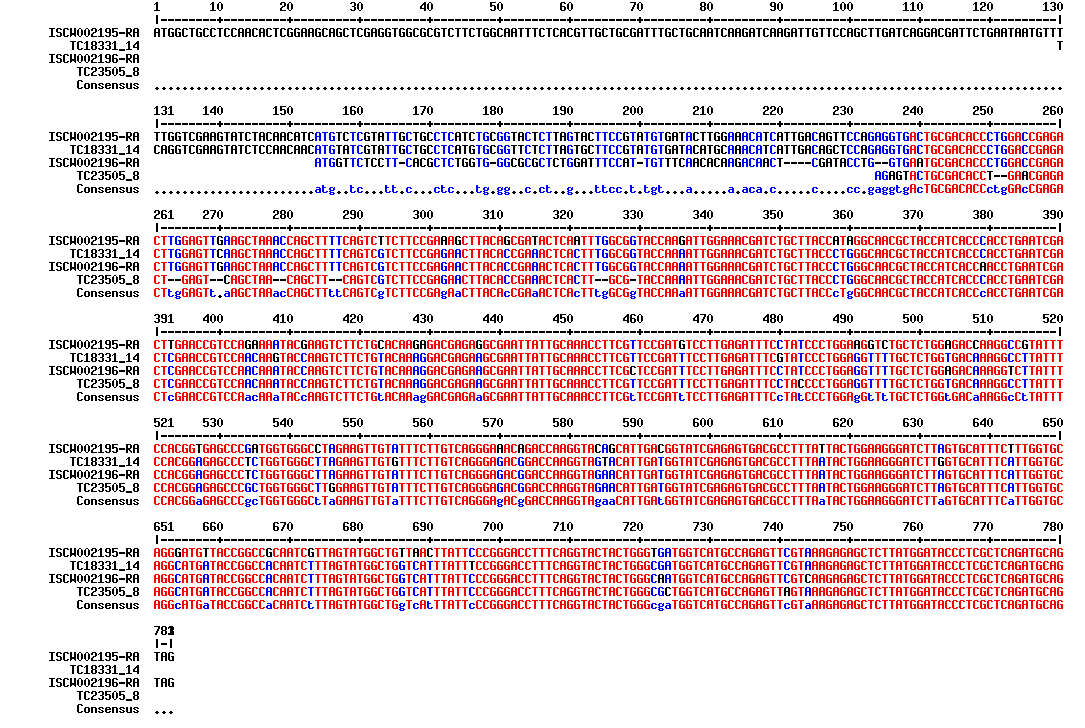


**Figure S5.**

**A.**

**
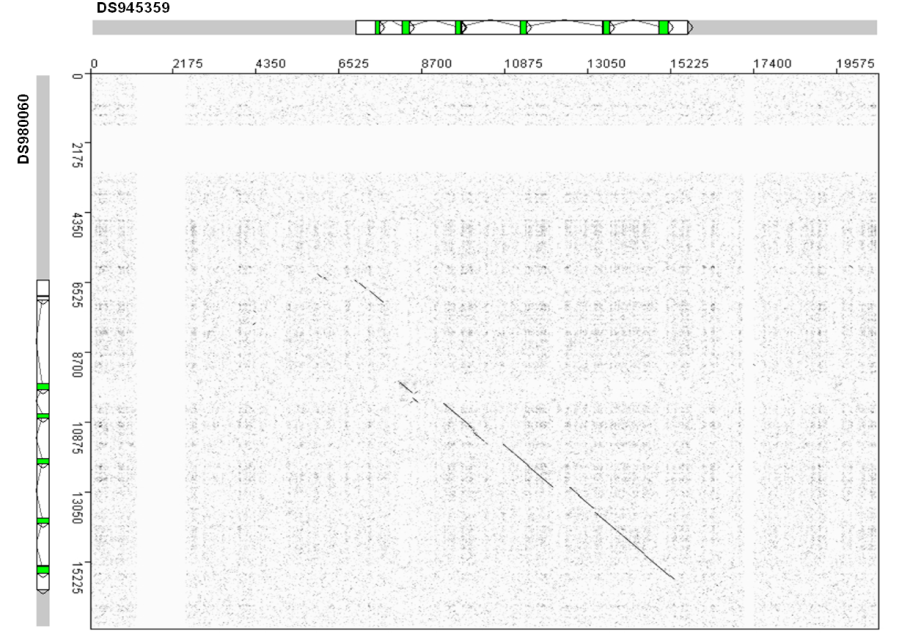
**

**B.**


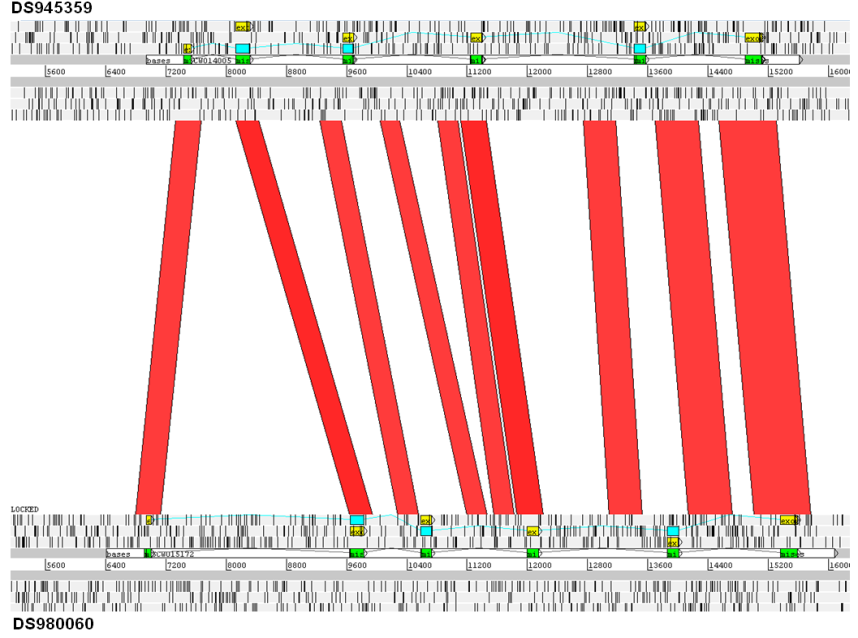


**Figure S6.**


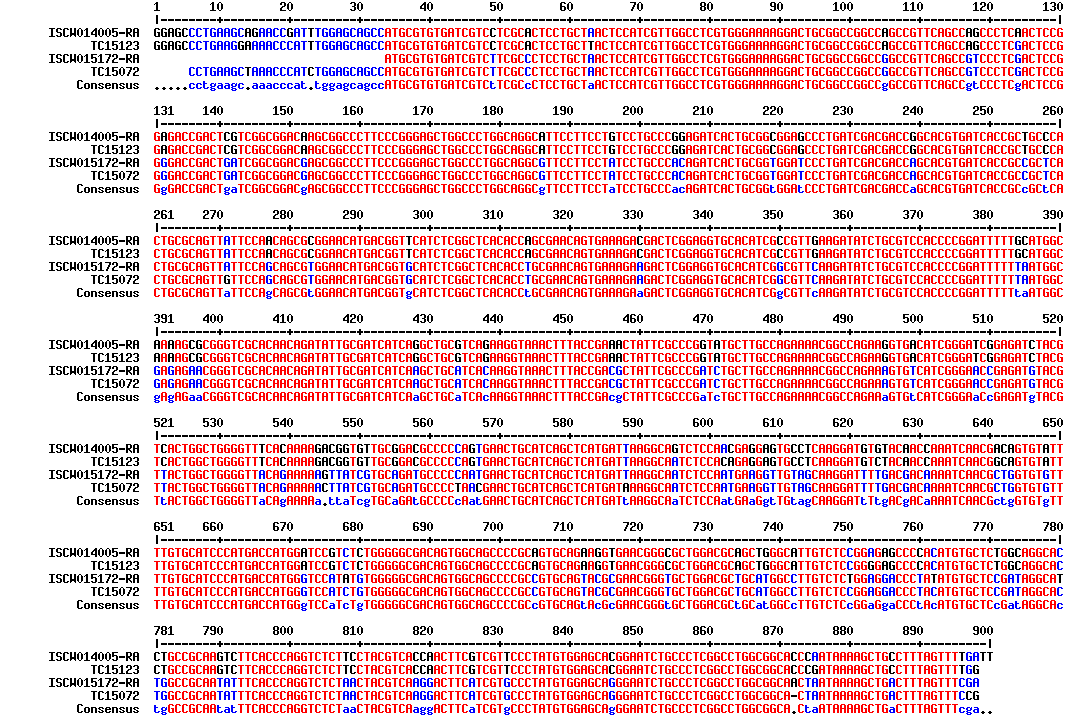


**Figure S7**.

**A.**

**
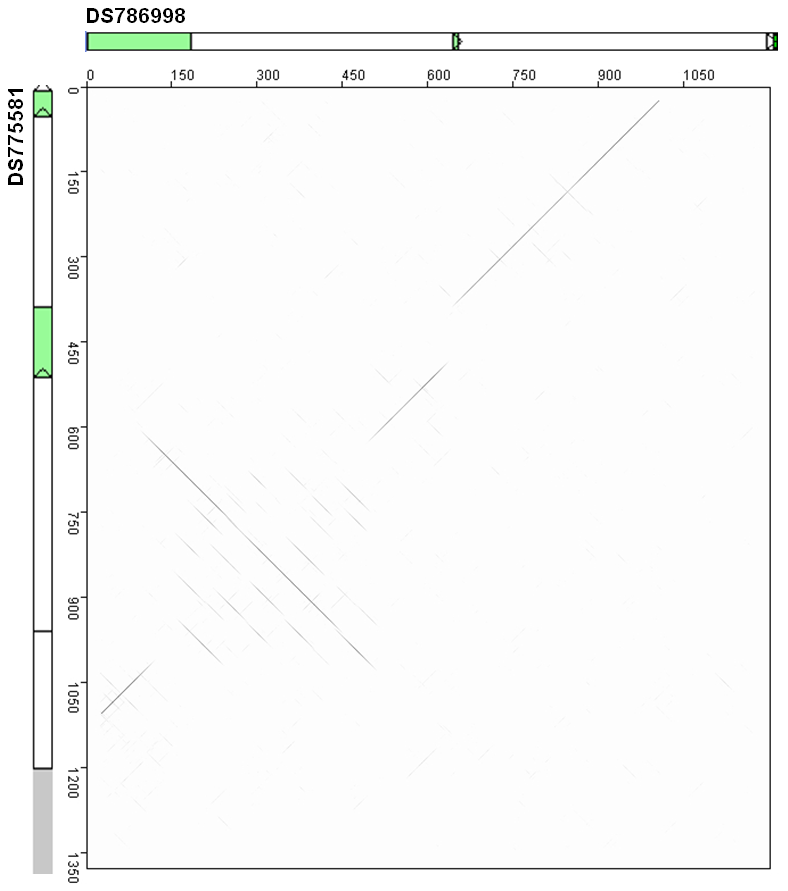
**

**B.**

**
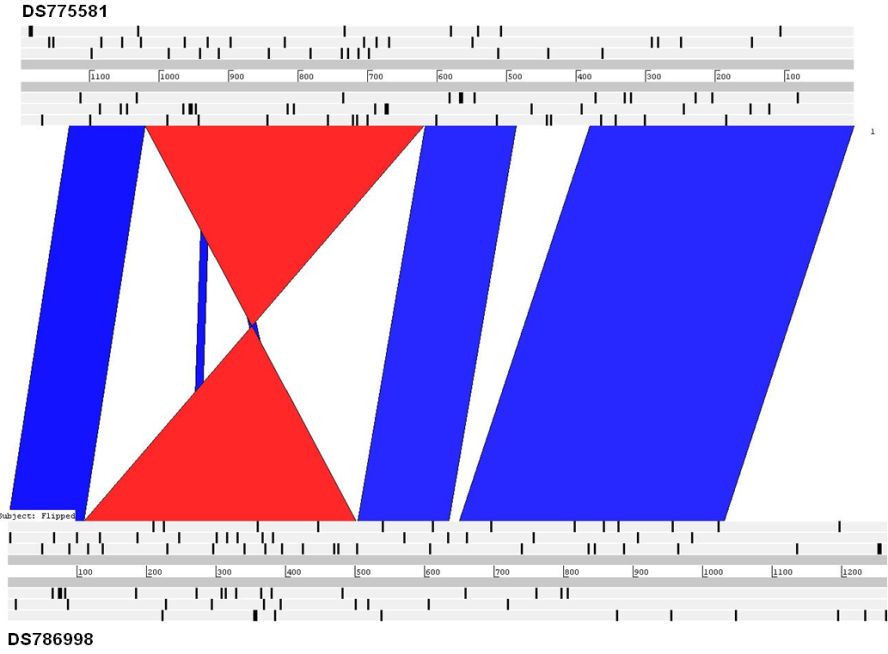
**

**Figure S8.**


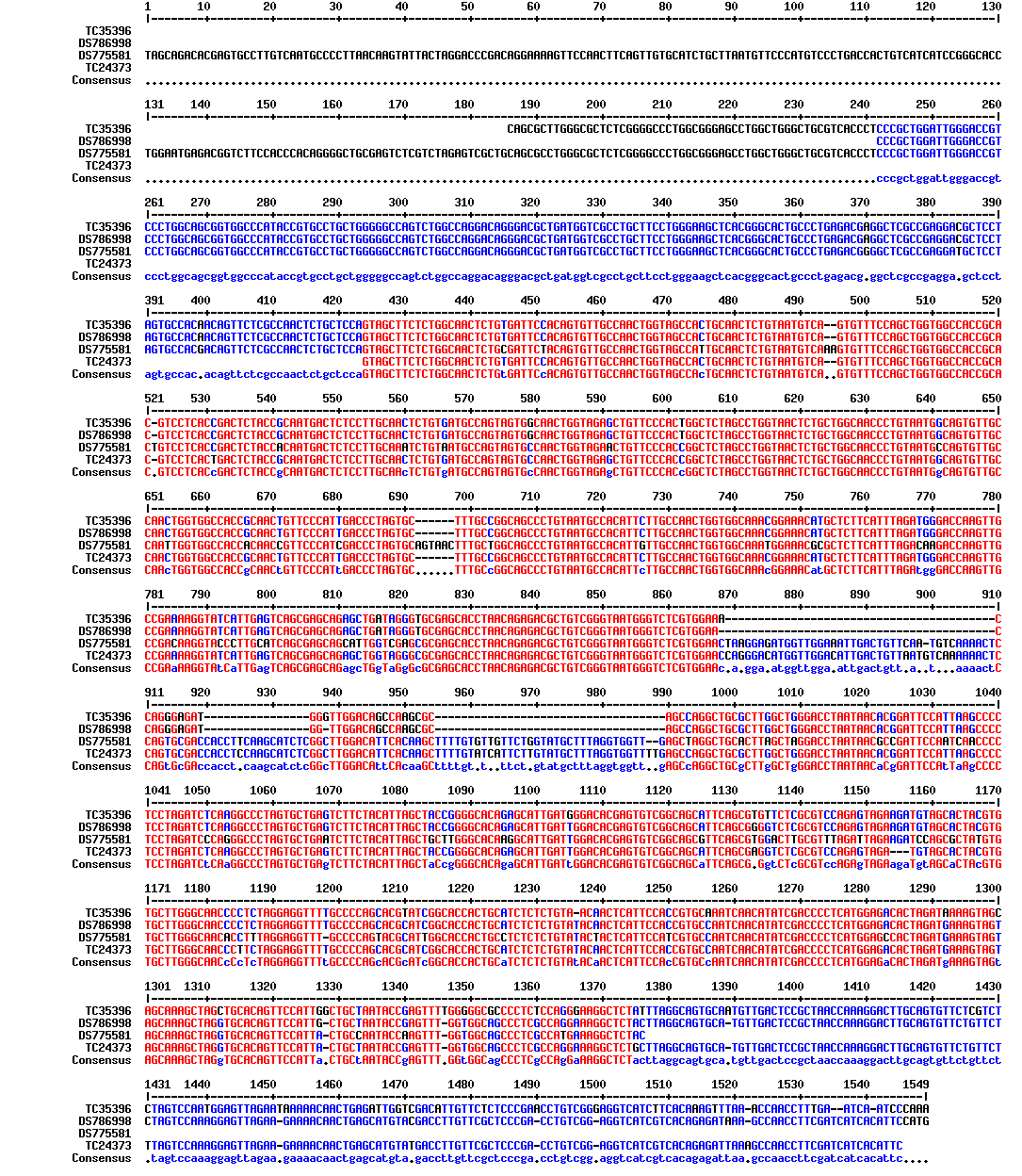


**Figure S9.**


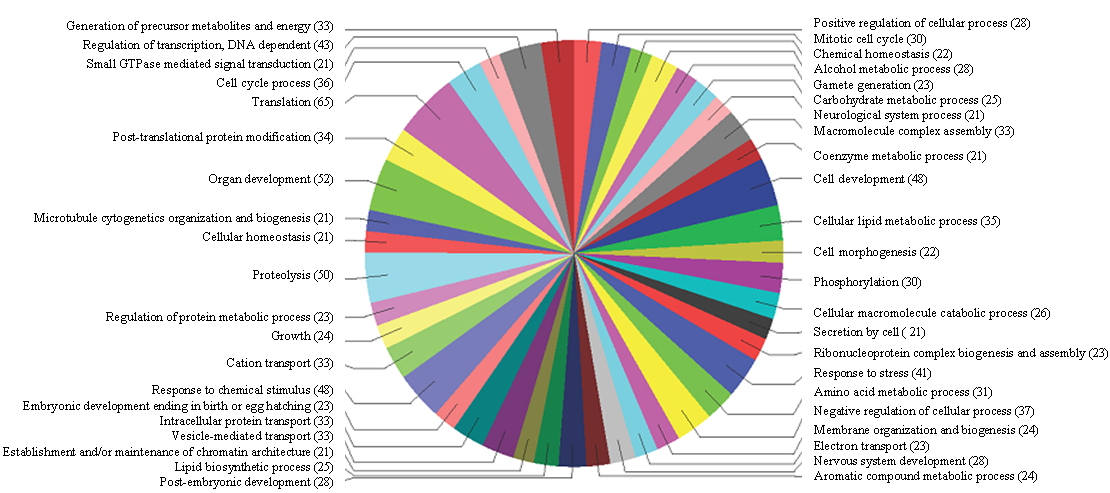


**Figure S10**.


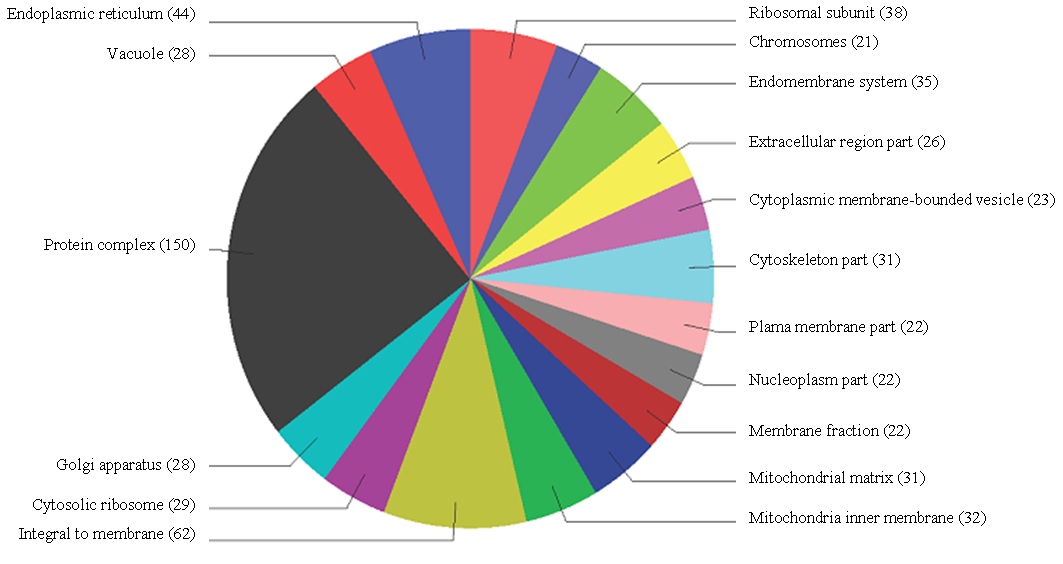


**Figure S11.**


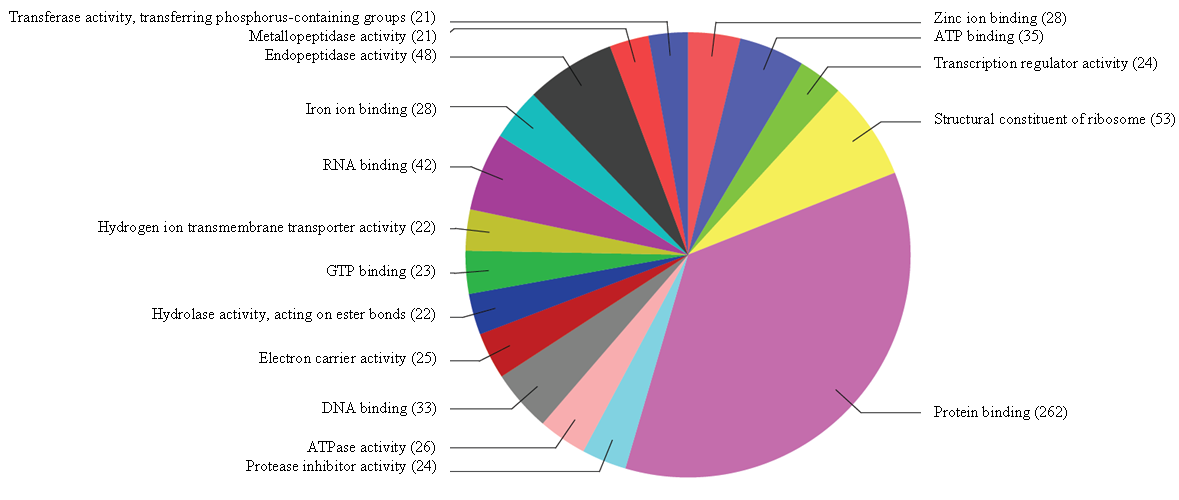


**Figure S12.**


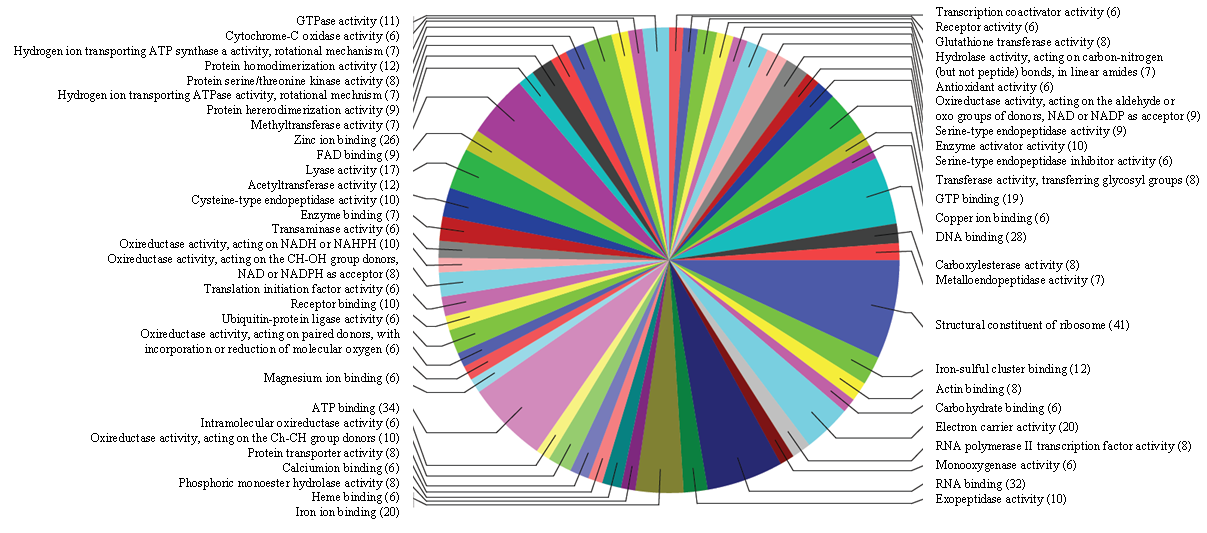


**Figure S13.**


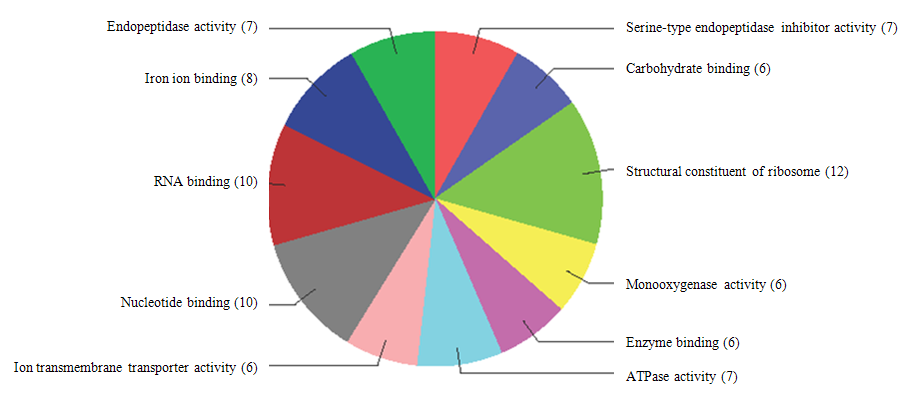


**Figure S14**.


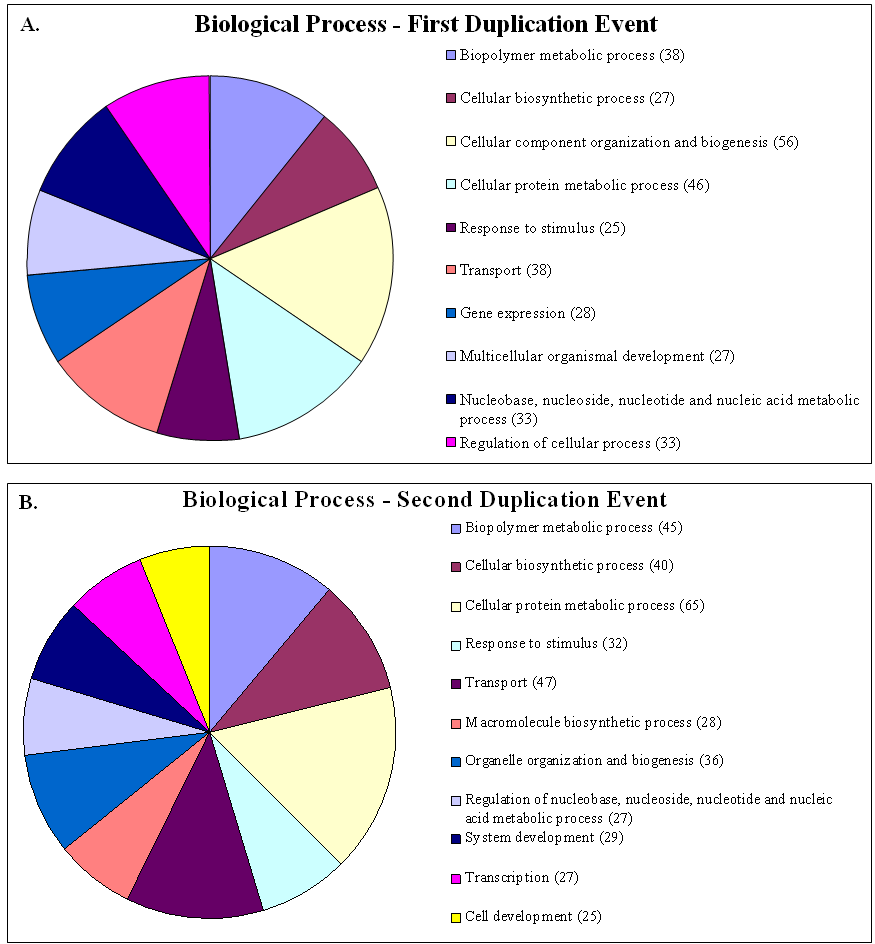


**Figure S15.**


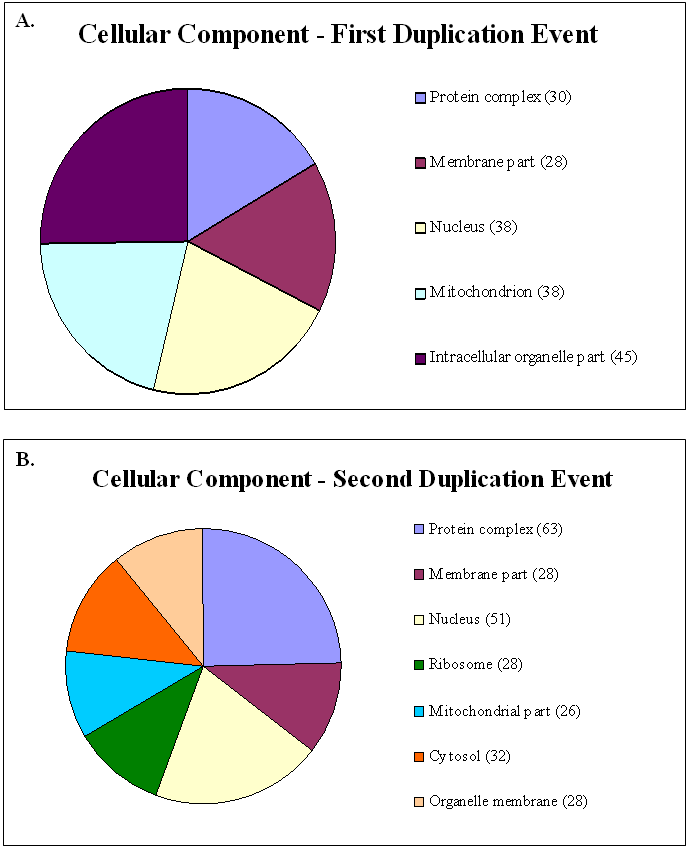


**Figure S16.**
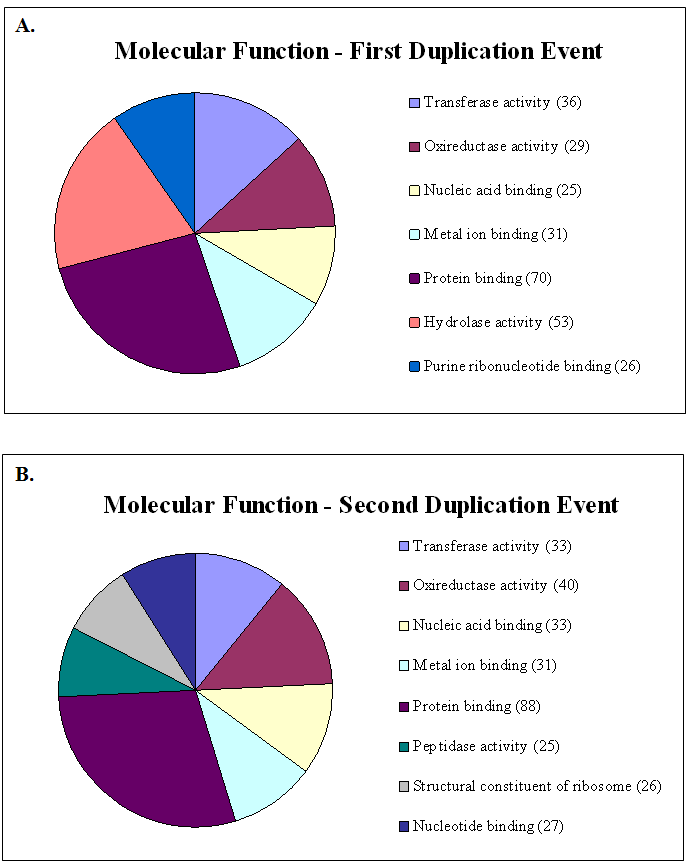


**Figure S17.**

**Table S1.** **Identification of paralogs in gene models (GMs) and tentative consensus (TC) sequences from four species of ixodid ticks.** Paralogs were identified by Vmatch using low, medium and high stringency parameters. The percentage of sequences identified as singletons or in clusters are shown. The number of clusters comprising exactly two sequences (clusters = 2) subsequently used in PAML analysis are shown in bold. Numbers in bold and parenthesis indicate the total number of clusters = 2 expressed as a percentage of the total number clusters identified by Vmatch.

| **Species Vmatch Stringency Level**  **Low^a^ Medium^b^ High^c^** | | | | |
| --- | --- | --- | --- | --- |
| ***Ixodes scapularis*** (Total number of gene models = 24,925) | | | | |
| Percentage of singletons^d^ | | 96.59% | 97.76% | 98.46% |
| Percentage of sequences in clusters^e^ | | 3.41% | 2.24% | 1.54% |
| Number of clusters^f^ | | 352 | 250 | 175 |
| **Number of clusters = 2^g^** | | **293 (83.2%)** | **216 (86.4%)** | **152 (86.9%)** |
| ***Ixodes scapularis*** (Total number of TCs = 20,901) | | | | |
| Percentage of sequences in singletons^d^ | 77.10% | | 86.40% | 92.46% |
| Percentage of sequences in clusters^e^ | 22.90% | | 13.60% | 7.54% |
| Number of clusters^f^ | 1,897 | | 1,169 | 682 |
| **Number of clusters = 2^g^** | **1,443 (76%)** | | **939 (80%)** | **593 (87%)** |
| ***Rhipicephalus microplus*** (Total number of TCs = 9,403) | | | | |
| Percentage of sequences in singletons^d^ | 94.58% | | 96.25% | 97.35% |
| Percentage of sequences in clusters^e^ | 5.42% | | 3.75% | 2.65% |
| Number of clusters^f^ | 237 | | 165 | 120 |
| **Number of clusters = 2^g^** | **209 (88%)** | | **146 (88.5%)** | **111 (92.5%)** |
| ***Rhipicephalus appendiculatus*** (Total number of TCs = 2,767) | | | | |
| Percentage of sequences in singletons^d^ | 92.12% | | 95.23% | 97% |
| Percentage of sequences in clusters^e^ | 7.88% | | 4.77% | 3% |
| Number of clusters^f^ | 90 | | 55 | 35 |
| **Number of clusters = 2^g^** | **65 (72.2%)** | | **38 (69%)** | **26 (74.3%)** |
| ***Amblyomma variegatum*** (Total number of TCs = 478) | | | | |
| Percentage of sequences in singletons^d^ | 91.63% | | 93.72% | 96.86% |
| Percentage of sequences in clusters^e^ | 8.37% | | 6.28% | 3.14% |
| Number of clusters^f^ | 17 | | 13 | 7 |
| **Number of clusters = 2^g^** | **12 (70.5%)** | | **9 (69.2%)** | **6 (85.7%)** |

**^a^**Low stringency: 75% of the smaller TC must match ≥ 50% of the larger TC of a paralog pair; **^b^**medium stringency: 85% of the smaller TC must match ≥ 70% of the larger TC of a paralog pair**; ^c^**high stringency: 95% of the smaller TC must match ≥ 80% of the larger TC of a paralog pair; **^d^**sequences that did not meet Vmatch minimum requirements were considered singletons; **^e^**similar sequences (paralogs or homologs) and possibly members of the same gene families; **^f^**total number of clusters identified by Vmatch; **^g^**total number of clusters = 2 identified by Vmatch and analyzed using PAML.

**Table S2.** **Paralogs identified in gene models (GMs) and the sampled transcriptome (TC sequences) of four species of ticks and analyzed by PAML.** The number of clusters = 2 identified by Vmatch (see Table S1) is expressed as a percentage of the total number of GMs or TC sequences**.**

| **Species** | **Vmatch Stringency Levels** | | |
| --- | --- | --- | --- |
|  | **Low^a^** | **Medium^b^** | **High^c^** |
| **Gene models (GMs)** | | | |
| ***Ixodes scapularis*** (Total number gene models = 24,925) | | | |
| Percentage of clusters = 2 | **2.35%** | **1.72%** | **1.22%** |
| **Tentative Consensus (TC) sequences** | | | |
| ***Ixodes scapularis*** (Total number TCs = 20,901) | | | |
| Percentage of clusters = 2 | **13.8%** | **.98%** | **5.67%** |
| ***Rhipicephalus microplus*** (Total number TCs = 9,403) | | | |
| Percentage of clusters = 2 | **4.44%** | **3.10%** | **2.36%** |
| ***Rhipicephalus appendiculatus*** (Total number TCs = 2,767) | | | |
| Percentage of clusters = 2 | **4.48%** | **2.74%** | **1.88%** |
| ***Amblyomma variegatum*** (Total number TCs = 478) | | | |
| Percentage of clusters = 2 | **5.02%** | **3.76%** | **2.51%** |

**^a^**Low stringency: 75% of of the smaller TC must match ≥ 50% of the larger TC of a paralog pair; **^b^**medium stringency: 85% of the smaller TC must match ≥ 70% of the larger TC of a paralog pair**; ^c^**high stringency: 95% of the smaller TC must match ≥ 80% of the larger TC of a paralog pair.

**Table S3. Dating of duplication events in *Ixodes scapularis* and *Rhipicephalus microplus* based on substitution rates from multiple species**. Calculations were made using median Ks values for each duplication event.

|  |  |  | ***Ixodes scapularis Rhipicephalus microplus***  **Event 1 Event 2 Event 1 Event 2 Event 3** | | | | |
| --- | --- | --- | --- | --- | --- | --- | --- |
|  | **Species** | **Substitution Rate** | **0.0124** | **0.092** | **0.0335** | **0.092** | **0.68** |
| Mammal | ***Homo sapiens^a^*** | 2.5 substitutions/site/MY | ~5MYA | ~37MYA | ~13MYA | ~37MYA | ~272MYA |
| Mammal | ***Mammals^b^*** | 3.5 substitutions/site /MY | ~3.5MYA | ~26MYA | ~10MYA | ~26MYA | ~194MYA |
| Plant | ***Arabidopsis thaliana^c^*** | 6.1 substitutions/site /MY | <1MYA | ~15.1MYA | ~5.5MYA | ~15.1MYA | ~111.5MYA |
| Insect | ***Drosophila melanogaster^d^*** | 16 substitutions/site /MY | <1MYA | ~5.7MYA | ~2.1MYA | ~5.7MYA | ~42.5MYA |
| Insect | ***Anopheles spp.^e^*** | 61 substitutions/site /MY | <1MYA | ~1.5MYA | <1MYA | ~1.5MYA | ~11.1MYA |
| Tick | ***Ornithodoros savigny^f^*** | 2.49 substitutions/site /MY | ~5MYA | ~37MYA | ~13.5MYA | ~37MYA | ~273MYA |
| Tick | ***Ornithodoros savigny^g^*** | 3.2 substitutions/site /MY | ~4MYA | ~29MYA | ~10.5MYA | ~29MYA | ~212MYA |
| Yeast | ***Saccharomyces cerevisiae^h^*** | 8.1 substitutions/site /MY | ~1.5MYA | ~11MYA | ~4MYA | ~11MYA | ~84MYA |

^a,c,d,h^Based on analysis of duplicated genes from sequenced genomes [16].

^b^Based on 47 protein coding sequence from primata and rodentia and considering the time of divergence between humans and rodents as 80 MYA [31].

^e^ Based on 157 cDNA and 40 EST sequences from *Anopheles gambiae* and *A. funestus* [33].

^f,g^Based on platelet aggregation inhibitor, disagregin and savignygrin gene sequences from soft ticks, and assuming the oldest tick fossil dated at 92 MYA [32].

MYA, million years ago.

**Table S4.** **Blast2GO functional annotation of *I. scapularis* putatively duplicated TC sequences under negative and positive selection and showing the number of genes assigned to common GO categories for the biological process, cellular component and molecular function categories (minimum of five sequences per pathway).**

| **Common gene ontology terms/pathways** | **Negative**  **selection** | **Positive**  **selection** |
| --- | --- | --- |
| **Biological Process** | **96 pathways** | **25 pathways** |
| Response to oxidative stress | 9 | 7 |
| Mitochondrion organization and biogenesis | 8 | 7 |
| Iron ion transport | 6 | 6 |
| Translation | 46 | 19 |
| **Cellular Component** | **35 pathways** | **10 pathways** |
| Chromatin | 6 | 6 |
| Cytosolic large ribosomal subunit | 8 | 6 |
| Microsome | 10 | 10 |
| Mitochondrial respiratory chain | 9 | 6 |
| **Molecular Function** | **55 pathways** | **11 pathways** |
| Structural constituent of ribosome | 41 | 12 |
| RNA binding | 32 | 10 |
| Iron ion binding | 20 | 8 |
| Enzyme binding | 7 | 6 |
| Serine-type endopeptidase inhibitor activity | 6 | 7 |
| Carbohydrate binding | 6 | 6 |
| Monooxygenase activity | 6 | 6 |

**Table S5.** **Blast2GO functional annotation of *I. scapularis* TC sequences assigned to the first and second duplication events and showing classification by biological process, cellular component and molecular function pathways (minimum of 25 sequences per pathway).**

| **GO term identification for the three gene ontology categories** | **First duplication event** | **Second duplication event** |
| --- | --- | --- |
| **Biological Process** |  |  |
| Gene expression | 28 | - |
| Multicellular organismal development | 27 | - |
| Cellular protein metabolic process | 46 | - |
| Cellular component organization and biogenesis | 56 | - |
| Regulation of cellular process | 33 | - |
| Nucleobase, nucleoside, nucleotide and nucleic acid metabolic process | 33 | - |
| Regulation of nucleobase, nucleoside, nucleotide and nucleic acid metabolic process | - | 27 |
| Transport | 38 | 47 |
| Cellular biosynthetic process | 27 | 40 |
| Response to stimulus | 25 | 32 |
| Biopolymer metabolic process | 38 | 45 |
| Macromolecule biosynthetic process | - | 28 |
| Organelle organization and biogenesis | - | 36 |
| System development | - | 29 |
| Transcription | - | 27 |
| Cell development | - | 25 |
| Cellular Protein metabolic process | - | 65 |
| **Cellular Component** |  |  |
| Intracellular organelle part | 45 |  |
| Protein complex | 30 | 63 |
| Membrane part | 28 | 28 |
| Nucleus | 38 | 51 |
| Mitochondrion | 38 | - |
| Mitochondrial part | - | 26 |
| Ribosome | - | 28 |
| Cytosol | - | 32 |
| Organelle membrane | - | 28 |
| **Molecular Function** |  |  |
| Purine ribonucleotide binding | 26 | - |
| Hydrolase activity | 53 | - |
| Transferase activity | 36 | 33 |
| Oxireductase activity | 29 | 40 |
| Protein binding | 70 | 88 |
| Metal ion binding | 31 | 31 |
| Nucleic acid binding | 25 | 33 |
| Peptidase activity | - | 25 |
| Structural constituent of ribosome | - | 26 |
| Nucleotide binding | - | 27 |

**Table S6. Blast2GO functional analysis of *Ixodes scapularis* putative duplicated TC sequences under positive selection showing annotation assigned to paralog pairs (molecular function category; minimum of 20 TC sequences for each GO annotation).**

| **Blast2GO Annotation: Molecular Function Category** | **No. Paralog Pairs** |
| --- | --- |
| 4-aminobutyrate aminotransferase/aminotransferase | 1 |
| 40s ribosomal protein s9 | 1 |
| Acetylcholinesterase | 1 |
| Amidohydrolase/aminoacylase 1-like 2 | 1 |
| AMP-dependent synthetase and ligase | 1 |
| ATPH^+^ mitochondrial subunit c3 (subunit 9) | 1 |
| Autophagocytosis protein | 1 |
| Beta-tubulin | 1 |
| Bifunctional GMP synthase glutamine amido-transferase protein | 1 |
| Bone morphogenetic protein 4 | 1 |
| C-type selectin-like (agap000929-pa) | 1 |
| Calmodulin | 2 |
| Catalase | 1 |
| Cathepsin D | 1 |
| Caveolin 1 | 1 |
| CD2 antigen (cytoplasmic tail) binding protein 2 | 1 |
| Chromobox homolog 3 | 1 |
| Chromosome 17 open reading frame 37 | 1 |
| Coiled-coil-helix-coiled-coil-helix domain containing 2 | 1 |
| Complement component q subcomponent binding protein | 1 |
| CRAL TRIO domain-containing protein | 1 |
| Cuticular protein | 1 |
| Cysteine/glycine-rich protein 2 | 1 |
| Cytochrome C | 1 |
| Cytochrome P450 | 2 |
| Delta protein | 1 |
| DNA replication licensing factor mcm7 | 1 |
| Eukaryotic translation initiation factor 1a domain containing | 1 |
| Eukaryotic translation initiation factor 3 subunit | 1 |
| Family with sequence similarity member a | 1 |
| Ferric-chelate reductase 1 | 1 |
| Ferritin | 1 |
| Four and a half LIM domains 3 | 1 |
| Glutathione peroxidase | 1 |
| Glutathione s-transferase D1 | 2 |
| Hexokinase | 1 |
| Histone h1 | 1 |
| Isocitrate dehydrogenase | 1 |
| kiaa1704 protein | 1 |
| Luciferase | 1 |
| Major facilitator superfamily protein | 1 |
| Methyltransferase type 11/methyltransferase like 7a | 1 |
| mgc80968 protein | 1 |
| Mitochondrial ribosomal | 1 |
| NADH dehydrogenase subunit i | 1 |
| o-sialoglycoprotein endopeptidase | 1 |
| Obstractor b/mucin-like peritrophin | 1 |
| Oligonucleotide oligosaccharide-binding fold containing 2a | 1 |
| Oligopeptidase A | 1 |
| Phenylalanine-4-hydroxylase | 1 |
| Polyubiquitin | 2 |
| Profilin | 1 |
| Protein, unknown function | 4 |
| Ribose 5-phosphate isomerase | 1 |
| Ribosomal protein L36/I9/S3/S5 | 4 |
| Secreted salivary protein | 1 |
| Selenoprotein t | 1 |
| Short-chain dehydrogenase reductase sdr/hydroxysteroid dehydrogenase like 2 | 1 |
| Thioredoxin | 1 |
| Thymosin isoform 1 | 1 |
| Tissue factor pathway inhibitor 2 | 2 |
| TNF receptor-associated factor 6 | 1 |
| TPR domain protein | 1 |
| Transcription factor | 1 |
| Translation initiation factor IF-2 subunit alpha | 1 |
| Ubiquinol-cytochrome c reductase binding protein | 1 |
| Vacuolar ATP synthase subunit e | 1 |
| Very low density lipoprotein receptor | 1 |
| Voltage-dependent anion channel 2 | 1 |
